# Reinforcement Learning Algorithms and Applications in Healthcare and Robotics: A Comprehensive and Systematic Review

**DOI:** 10.3390/s24082461

**Published:** 2024-04-11

**Authors:** Mokhaled N. A. Al-Hamadani, Mohammed A. Fadhel, Laith Alzubaidi, Harangi Balazs

**Affiliations:** 1 Department of Data Science and Visualization, Faculty of Informatics, University of Debrecen, H-4032 Debrecen, Hungary; harangi.balazs@inf.unideb.hu; 2 Doctoral School of Informatics, University of Debrecen, H-4032 Debrecen, Hungary; 3 Department of Electronic Techniques, Technical Institute/Alhawija, Northern Technical University, 36001 Kirkuk, Iraq; 4 Research and Development Department, Akunah Company, Brisbane, QLD 4120, Australia; mohammed.a.fadhel@uoitc.edu.iq (M.A.F.); l.alzubaidi@qut.edu.au (L.A.); 5 School of Mechanical, Medical, and Process Engineering, Queensland University of Technology, Brisbane, QLD 4000, Australia; 6 Centre for Data Science, Queensland University of Technology, Brisbane, QLD 4000, Australia

**Keywords:** reinforcement learning, dynamic programming, Monte Carlo, temporal difference, cell growth, object grasping and manipulation

## Abstract

Reinforcement learning (RL) has emerged as a dynamic and transformative paradigm in artificial intelligence, offering the promise of intelligent decision-making in complex and dynamic environments. This unique feature enables RL to address sequential decision-making problems with simultaneous sampling, evaluation, and feedback. As a result, RL techniques have become suitable candidates for developing powerful solutions in various domains. In this study, we present a comprehensive and systematic review of RL algorithms and applications. This review commences with an exploration of the foundations of RL and proceeds to examine each algorithm in detail, concluding with a comparative analysis of RL algorithms based on several criteria. This review then extends to two key applications of RL: robotics and healthcare. In robotics manipulation, RL enhances precision and adaptability in tasks such as object grasping and autonomous learning. In healthcare, this review turns its focus to the realm of cell growth problems, clarifying how RL has provided a data-driven approach for optimizing the growth of cell cultures and the development of therapeutic solutions. This review offers a comprehensive overview, shedding light on the evolving landscape of RL and its potential in two diverse yet interconnected fields.

## 1. Introduction

Today, artificial intelligence (AI) is present in all areas of life and helps to operate in an increasingly dynamic way in line with its evolving capabilities. In the pursuit of creating machines that can think and learn autonomously, without human intervention, we have reached the crossroads of artificial intelligence (AI) and reinforcement learning (RL) [1,2]. As Alan Turing once said, “A machine that could learn from its own mistakes, now there’s a thought” [3]. Therefore, this “thought” has evolved into reality when RL illuminates the path to intelligent machines capable of autonomous decision-making and complex problem-solving [4].

RL is one of the machine learning branches that has gained tremendous attention in recent years [5]. RL’s goal is to allow machines to learn through trial and error, which surpasses all the other methods. More precisely, RL agents learn to map the optimal situations to actions and this is what is called optimal policy. These actions have to obtain the highest reward. Although these actions may not affect the current reward, they may affect the subsequent rewards. Therefore, the reinforcement learning problem features can be distinguished by the actions and the subsequent outcomes of these actions which could include the reward signals [6]. Moreover, RL tries to imitate the mechanism of human learning, which is considered to be a step towards artificial intelligence [7].

In reinforcement learning problems, an agent engages in interactions with its environment. The environment, in turn, provides rewards and new states based on the actions of the agent. In reinforcement learning, the agent is not explicitly taught what to do; instead, it is presented with rewards based on its actions. The primary aim of the agent is to maximize its overall reward accumulation throughout time by executing actions that yield positive rewards and refraining from actions that yield negative rewards.

**Figure 1:**
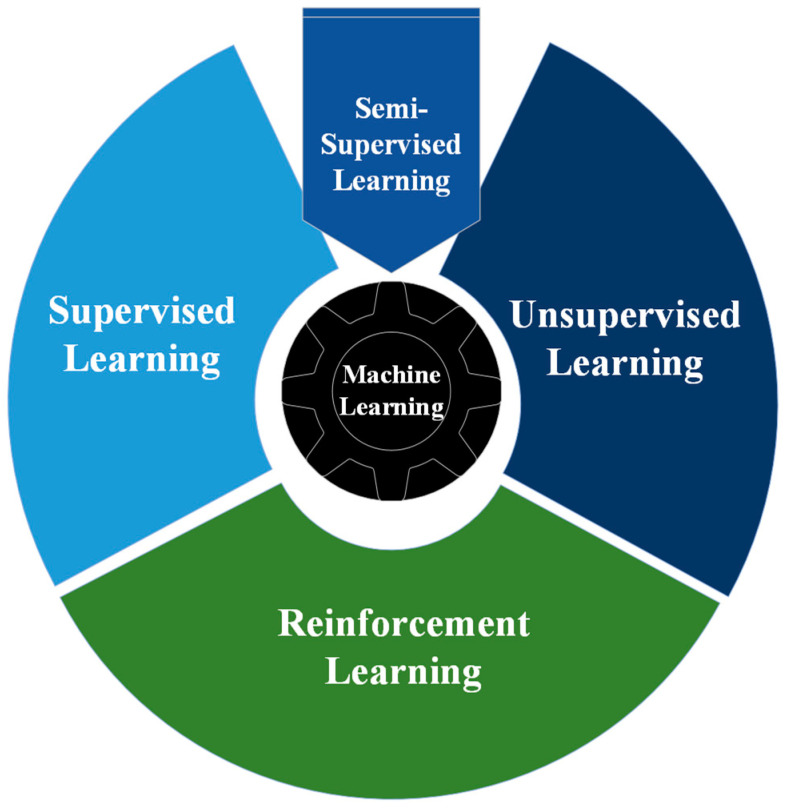
Machine learning branches.

Reinforcement learning differs from other categories of machine learning, namely supervised, unsupervised, and semi-supervised learning. RL learns through a process of trial and error that aims to maximize the cumulative reward of an action in any given environment. Traditional machine learning branches can be specified as shown in Figure 1. Supervised learning: This method involves learning from a training dataset labeled with desired results [8,9]. It is the most common learning approach in the machine learning field. The objective is to generalize the model so that it can perform effectively on data not present in the training set. Unsupervised learning: This method operates with unlabeled data, unlike supervised learning. It is more challenging as it lacks actual labels for comparison. The model attempts to learn the characteristics of the data and then clusters these data samples based on their similarities [10]. Semi-supervised learning: This type is a combination of supervised and unsupervised learning. The dataset is partially labeled, while the rest is unlabeled data [11]. The goal is to cluster a large amount of the unlabeled data using unsupervised learning techniques and then label them based on supervised techniques.

Reinforcement learning presents several distinctive challenges that set it apart from other machine learning approaches. These challenges involve aspects like managing the trade-off between exploration and exploitation to maximize the cumulative reward and addressing the broader issue of an agent interacting with an unfamiliar environment [6].

Before delving deeply into our review paper, it is essential to present recent survey and review papers that are related to reinforcement learning in robotics manipulation and healthcare (cell growth problems). Table 1 summarizes their contributions and highlights the differences between their work and ours. In [12], a systematic review of deep reinforcement learning (DRL)-based manipulation is provided. The study comprehensively analyzes 286 articles, covering key topics such as grasping in clutter, sim-to-real, learning from demonstration, and other aspects related to object manipulation. The review explores strategies for data collection, the selection of models, and their learning efficiency. Additionally, the authors discuss applications, limitations, challenges, and future research directions in object grasping using DRL. While our work in the robotics section broadly covers object manipulation using RL approaches, this study specifically focuses on DRL, offering a nuanced examination of approaches and their limitations. In [13], the authors conduct an extensive examination of deep reinforcement learning algorithms applied to the field of robotic manipulation. This review offers a foundational understanding of reinforcement learning and subsequently places a specific focus on deep reinforcement learning (DRL) algorithms. It explores their application in tackling the challenges associated with learning manipulation tasks, including grasping, sim-to-real transitions, reward engineering, and both value-based and policy-based techniques over the last seven years. The article also delves into prominent challenges in this field, such as enhancing sample efficiency and achieving real-time control, among others. Nevertheless, it is worth noting that this study does not offer a detailed analysis of the results of these techniques, whether in simulation or real-world scenarios, as is undertaken in the present review. In [14], the authors aim to provide an extensive survey of RL applications to various decision-making problems in healthcare. The article commences with a foundational overview of RL and its associated techniques. It then delves into the utilization of these techniques in healthcare applications, encompassing dynamic treatment regimes, automated medical diagnosis in structured and unstructured data, and other healthcare domains, including health resource scheduling and allocation, as well as drug discovery and development. The authors conclude their work by emphasizing the most significant challenges and open research problems while indicating potential directions for future work. Our work distinguishes itself from this study in terms of its specific focus on RL techniques and healthcare applications, which take a particular direction concerning cell growth problems. Finally, in [15], the authors discuss the impact of RL in the healthcare sector. The study offers a comprehensive review of RL and its algorithms used in healthcare applications. It highlights healthcare applications grouped into seven categories, starting with precision medicine and concluding with health management systems, showcasing recent studies in these areas. Moreover, the authors employ a statistical analysis of the articles used to illustrate the distribution of articles concerning various terms, including category and approach. Lastly, the study explores the strengths and challenges associated with the application of RL approaches in the healthcare field.

Therefore, this study distinguishes itself from the above review/survey papers by employing a combination of comprehensive and systematic reviews. It emphasizes the following key aspects:

- This study offers a fundamental overview of reinforcement learning and its algorithms.
- It conducts a comparative analysis of RL algorithms based on various criteria.
- The applications covered in this review encompass both the robotics and healthcare sectors, with specific topics selected for each application. In the realm of robotics, object manipulation and grasping have garnered considerable attention due to their pivotal roles in a wide range of fields, from industrial automation to healthcare. Conversely, for healthcare, cell growth problems were chosen as a focus area. This topic is of increasing interest due to its significance in optimizing cell culture conditions, advancing drug discovery, and enhancing our understanding of cellular behavior, among other potential benefits.

The remainder of this paper is organized as follows: Section 2 outlines the methodology employed in this study. Section 3 illustrates the comprehensive science mapping analysis for all the references used in this review. Section 4 introduces RL and its algorithms. Section 5 reviews recent articles on two RL applications, elucidating their challenges and limitations. Finally, Section 6 contains the conclusion and future directions of this review.

## 2. Methodology

**Figure 2:**
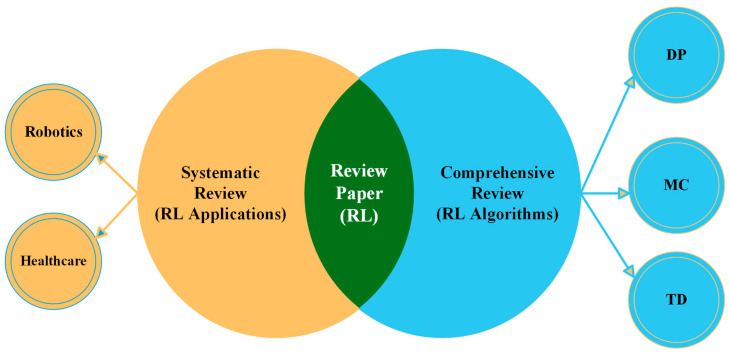
Structure layout of our review paper.

This review paper is structured into two distinct sections, as illustrated in Figure 2. The first part is a comprehensive review, which is a traditional literature review with the objective of offering a broad overview of the existing literature on a specific topic or subject [16]. This type of review, also known as a literature review or narrative review, can encompass various sources, including peer-reviewed original research, systematic reviews, meta-analyses, books, PhD dissertations, and non-peer-reviewed articles [17]. Comprehensive literature reviews (CLRs) have several advantages. They are generally easier to conduct than systematic literature reviews (SLRs) as they rely on the authors’ intuition and experience, allowing for some subjectivity. Additionally, CLRs are shaped by the authors’ assumptions and biases, which they can openly acknowledge and discuss [18]. Consequently, the initial part of this review offers a highly comprehensive introduction to reinforcement learning and its components. Subsequently, this review delves into the specifics of RL algorithms, highlighting their differences based on various criteria.

The second part of this paper is a systematic literature review (SLR), which follows a rigorous and structured approach to provide answers to specific research questions or address particular problems [19]. Systematic reviews are commonly employed to confirm or refute whether current practices are grounded in relevant evidence and to assess the quality of that evidence on a specific topic [20] An SLR is an evaluation of the existing literature that adheres to a methodical, clear, and replicable approach during the search process [17]. This methodology involves a well-defined research question, predefined inclusion and exclusion criteria, and a comprehensive search of relevant databases, often restricted to peer-reviewed research articles meeting specific quality and relevance criteria [21]. What sets SLRs apart from CLRs is their structured, replicable, and transparent process, guided by a predefined protocol. Consequently, the remainder of the paper, focusing exclusively on RL applications, including those in robotics and healthcare, adheres to the systematic review process. This approach involves concentrating on specific topics and analyzing articles to generate evidence and answers for those specific questions or topics.

**Figure 3:**
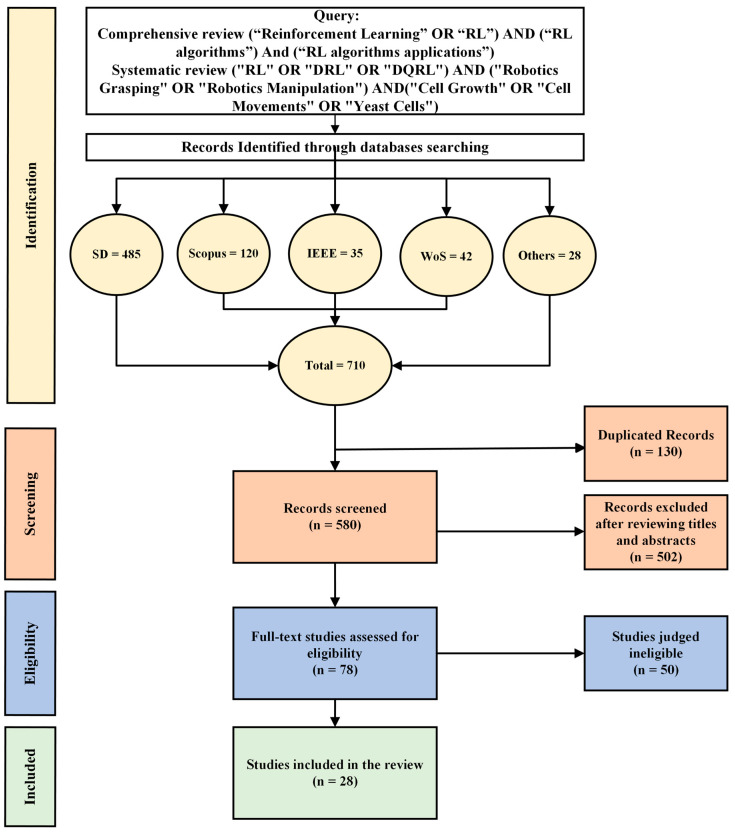
An outline of the approach of selecting studies, search query, and inclusion criteria.

This study has collected articles following the systematic review procedures outlined in Figure 3 [22,23]. The PRISMA statement, which is known as Preferred Reporting Items for Systematic Reviews and Meta-Analysis, was adopted to carry out a systematic review of the literature. The review process in this study involved queries from multiple reputable databases, including Science Direct (SD), IEEE Xplore digital library (IEEE), Web of Science (WoS), and Scopus. Additionally, other papers, PhD dissertations, and books were selected from ArXiv, PubMed, ProQuest, and MIT Press, respectively. The search for publications encompassed all scientific productions up to December 2023.

### 2.1. Search Strategy

A comprehensive review was performed of the articles in the mentioned databases above. This article employed a Boolean query (conclude OR, AND) to establish a connection between the keywords for each part of the review. The search strategy for this comprehensive review includes this query (“Reinforcement Learning” OR “RL”) AND (“RL algorithms”) AND (“RL algorithms applications”). The search strategy for this systematic review incorporates the following query: (“RL” OR “DRL” OR “DQRL”) AND (“Robotics Grasping” OR “Robotics Manipulation”). The other query is identical, with the only difference being the replacement of (“Robotics” with “Cell Growth” OR “Cell Movements” OR “Yeast Cells”). The collected articles for this systematic review from databases were published from 2022 to December 2023.

### 2.2. Inclusion and Exclusion Criteria

The inclusion criteria for this study encompass articles written in the English language and presented to reputable journals and conferences. The primary focus of this study involves reinforcement learning (RL) and RL algorithms, with specific attention to applications in robotics and healthcare. In healthcare, we concentrate on issues related to cell growth in yeast and mammalian cells. Conversely, the exclusion criteria encompass articles not composed in the English language and those lacking clear descriptions of methods, strategies, tools, and approaches for utilizing RL in these applications.

### 2.3. Study Selection

The selection process has been conducted based on the PRISMA statement for conducting a systematic review of the literature [22,23]. The articles were collected using Mendeley software (v2.92.0) to scan titles and abstracts. Research articles meeting the inclusion criteria mentioned in Section 2.2 were fully read by the authors.

In the initial search, a total of 710 studies were obtained, comprising 485 from SD, 120 from Scopus, 35 from IEEE, 42 from WoS, and 28 from other sources. The included articles in this study were disseminated starting from the initiation of scientific production until December 2023. Approximately 130 duplicate articles were eliminated from the databases, reducing the total number of articles to 580 contributions. During the screening phase of the titles and abstracts, 502 articles were excluded. In the full-text phase, 50 studies were deemed irrelevant, and the remaining 28 articles were selected according to the inclusion criteria. The following section explores the utilization of various bibliometric methods for analyzing the selected studies.

## 3. Comprehensive Science Mapping Analysis

The proliferation of contributions and the implementation of practical research made the task of identifying crucial evidence from previous studies more arduous. Keeping up with the literature became a considerable problem due to the extensive flow of practical and theoretical contributions. A number of scholars have proposed using the PRISMA methodology to restructure the results of prior research, condense issues, and pinpoint promising areas for further investigation. Systematic reviews, on the other hand, have the objective of broadening the knowledge base, improving the study design, and consolidating the findings of the literature. Nevertheless, systematic reviews encounter challenges regarding their credibility and impartiality since they depend on the authors’ perspective to rearrange the conclusions of prior investigations. In order to enhance the clarity in summarizing the findings of prior research, a number of studies have proposed techniques for carrying out a more thorough scientific mapping analysis using R-tool and VOSviewer [24]. The bibliometric technique yields definitive outcomes, investigates areas of study that have not been addressed, and presents the findings of the existing literature with a high degree of dependability and clarity. Moreover, the tools given in this context do not need significant expertise and are regarded as open source. Consequently, this research has used the bibliometric technique, which will be thoroughly explained in the subsequent subsections. The science mapping analysis demonstrates notable patterns of expansion in the field of reinforcement learning. The annual publication tally increased consistently, albeit with fluctuations, from one in 1950 to thirteen in 2023. Reputable publications such as Proceedings of the National Academy of Sciences received numerous citations. The literature is predominantly characterized by the prevalence of usual terms like “reinforcement learning” and “machine learning”. The word cloud emphasizes critical concepts such as ‘control’ and ‘algorithms’. Through the identification of clusters of related terms, co-occurrence network analysis reveals both fundamental and specialized concepts. In general, the analysis offers significant insights into the dynamic field of reinforcement learning investigation.

### 3.1. Annual Scientific Production

**Figure 4:**
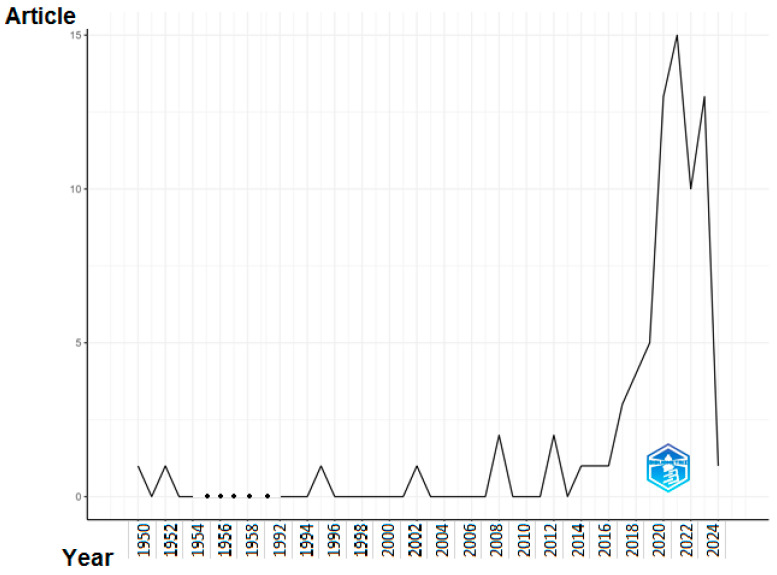
Annual Scientific Production.

The discipline of reinforcement learning has observed significant advancements in the last decade. Figure 4 displays the yearly scientific output, measured by the number of papers, in a specific study domain spanning from 1950 to 2024. The data may be analyzed and examined using the following methods:

General trajectory: The general trajectory shows a consistent increase, as the annual publication count has risen from 1 in 1950 to 13 in 2023. Nevertheless, the data show significant variations, with some years seeing a decline in output.

Early years: During the first time of the table’s existence (1950–1970), there was a minimal amount of scholarly output, with a mere four publications published in total. Indications point to the fact that the scientific area was in its nascent phase of advancement during this period.

Growth era: The period spanning from 1971 to 1995 had a substantial surge in scientific output, with a total of six publications produced throughout this timeframe. This indicates that the study area was starting to acquire momentum and receive more attention from scientists.

In the years spanning from 1996 to 2024, there has been a notable increase in scientific productivity, resulting in the publication of 54 publications within this time frame. These findings indicate that the research area has reached a state of maturity and is flourishing.

**Figure 5:**
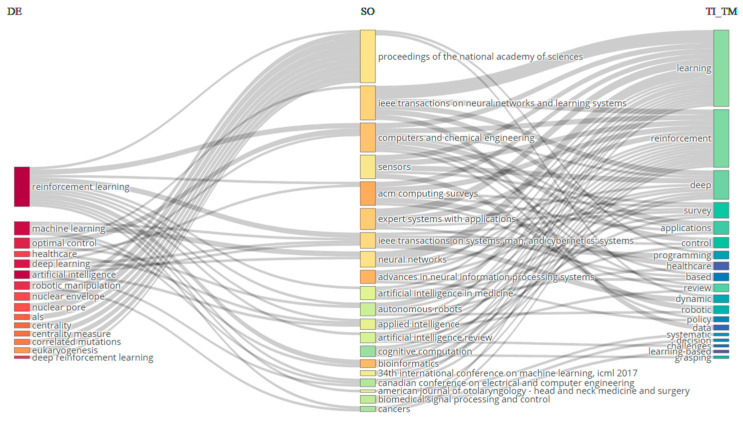
Three-field plot: left (SO), middle (CR_SO), and right (DE).

A three-field plot is a graphical representation used to exhibit data involving three variables. In this specific instance, the left field corresponds to keywords (DE), the center field corresponds to sources (SO), and the right field corresponds to Title (TI_TM). The plot is often used for the analysis of the interrelationships among the three parameters (refer to Figure 3). The analysis, identified in the middle sector (SO) of Figure 5, reveals that the Proceedings of the National Academy of Sciences, IEEE Transactions on Neural Networks and Learning Systems, and Computers and Chemical Engineering have received the highest number of citations from the sources (TI_TM) situated on the left side. The Proceedings of the National Academy of Sciences is the preeminent source that specifically addresses the subject of reinforcement learning. In addition, it is acknowledged in the field of DE that the most frequently used keywords across all categories are ‘reinforcement learning’, ‘machine learning’, ‘optimal control’, ‘healthcare’, ‘deep learning’, and ‘artificial intelligence’. These keywords are also commonly found in the journals listed in the middle field (SO).

### 3.2. Word Cloud

**Figure 6:**
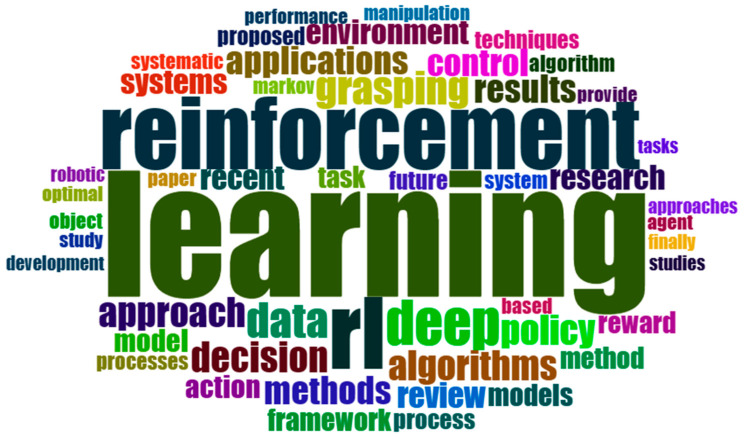
World cloud.

The use of word cloud has facilitated the identification of the most recurrent and crucial terms in previous research. Figure 6 compiles the essential keywords extracted from previous research results to provide a comprehensive overview and restructure the existing knowledge.

The word cloud visually displays the predominant phrases used in a scientific work pertaining to reinforcement learning (RL). The dominant words include reinforcement, learning, algorithms, methods, control, data, decision, deep, environment, and model. This study primarily emphasizes the advancement and utilization of RL algorithms and methodologies for managing robots and other systems in intricate contexts.

This study also examines the use of reinforcement learning (RL) in the domains of decision-making and task planning. Indicatively, this article pertains to a broad spectrum of applications, including robotics and healthcare.

Based on the word cloud and table, it can be inferred that this article provides a thorough examination of the current advancements in RL. This publication is expected to captivate scholars and practitioners in the area of RL, as well as anyone intrigued by the capacity of RL to address practical issues.

### 3.3. Co-Occurrence

**Figure 7:**
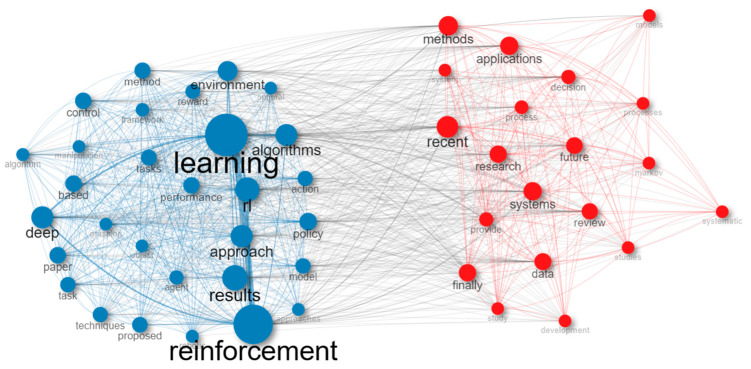
Co-occurrence network.

A co-occurrence network is another method used in bibliometric analysis. Previous research studies have identified common terms and analyzed them using a semantic network. This network offers valuable insights to professionals, policymakers, and scholars on the conceptual framework of a certain area. Figure 7 specifically presents data on a co-occurrence network that is constructed using the names of reinforcement learning methods and applications.

The co-occurrence network Table 2 displays the associations among the most prevalent phrases in a scholarly publication on reinforcement learning (RL). The nodes in the table correspond to the terms, while the edges reflect the connections between the terms. The words are categorized into clusters according to their interconnections. The most prominent cluster shown in Figure 7 comprises the phrases reinforcement learning, learning, algorithms, methods, and control. This cluster embodies the fundamental principles of reinforcement learning. The phrases data, decision, applications, techniques, and review are intricately interconnected with these fundamental principles. The additional clusters shown in Figure 6 correspond to more specialized facets of reinforcement learning. For instance, the cluster including the phrases grasping, manipulation, and robotic signifies the use of reinforcement learning (RL) in the context of robotics applications. The cluster including the phrases deep learning, policy, and reward signifies the use of RL for deep reinforcement learning. In general, the co-occurrence network table offers a comprehensive summary of the main ideas and connections in the scientific article on RL. Table 2 serves the purpose of discerning the important words in the document, together with the interconnections among these phrases. The co-occurrence network table serves as a tool to detect novel research prospects in the field of reinforcement learning (RL) and to pinpoint regions that need further investigation.

## 4. Reinforcement Learning (RL)

Reinforcement learning has emerged from two essential fields: psychology, inspiring trial-and-error search; and optimal control, using value functions and dynamic programming [6,25]. The first field has been derived from the animal psychology of trial-and-error learning. The concept of this learning started with Edward Thorndike [26]. Thorndike referred to this principle as the law of effect, describing how reinforcing events influence the trajectory of selected actions. In other words, it implies that the agent should take actions that yield the best rewards instead of facing punishment because the objective of RL is to maximize the cumulative reward through the concept of trial and error. In the second field, the ‘optimal control’ problem was proposed to devise a controller that minimizes a measure of a dynamical system over a duration of time [27]. The optimal control problem was introduced in the late 1950s for the same reasons mentioned earlier. Richard Bellman developed one of the techniques for this problem, creating an equation that utilizes the state of a dynamic system and a value function, widely recognized as the Bellman equation, which serves to define a functional equation [28]. The Bellman equation represents the long-term reward for executing a specific action corresponding to a particular state of the environment. This equation will be subjected to an elaborate analysis in Section 4.2.1. Furthermore, in 1957, Richard Bellman extended the work of Hamilton and Jacobi to solve optimal control problems using the Bellman equation, giving rise to what is known as dynamic programming [29]. Later in the same year, Bellman introduced Markov Decision Processes (MDPs), a discrete stochastic version of the optimal control problem. In 1960, Ronald Howard established policy iteration for Markov Decision Processes. Consequently, these two fields played a pivotal role in the development of the modern field of reinforcement learning. For more details about the history of RL, please refer to [6].

### 4.1. Reinforcement Learning Components

**Figure 8:**
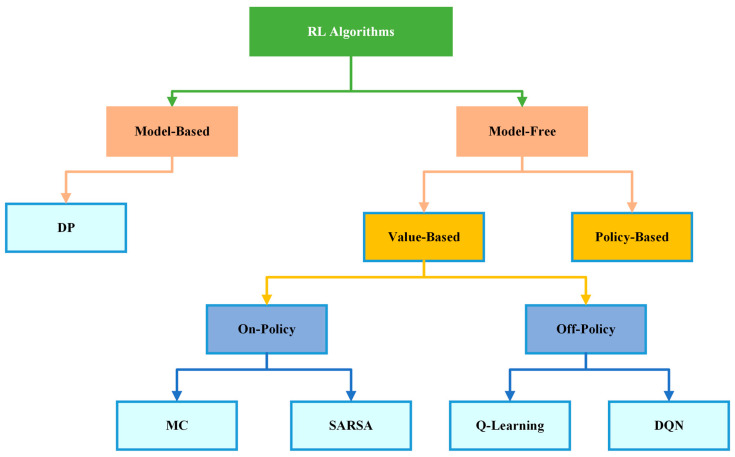
Taxonomy of reinforcement learning algorithms.

As previously stated, reinforcement learning is a subfield of machine learning that teaches an agent to take an action in an unknown environment that maximizes the reward over time. In other words, the purpose of RL is to determine how an agent should take an actions in an environment to maximize the cumulative reward. Accordingly, we noticed that RL has some essential components such as an *agent*, the program or algorithm that one trains, or what is called a learner or decision maker in RL, which aims to achieve a specific goal; an *environment*, which refers to the real-world problems or simulated environment in which an agent takes an action or interacts; *action*(A), the move that an agent makes in the environment which causes a change in the status; and a *reward*(R), which refers to the evaluation of the agent by taking an action that could give a positive or negative reward. Moreover, it has some other important components such as *state* (S), the place that an agent is located in in the environment; *episode*, the whole training process phase; *step* (t), as each operation in an episode is a step time; and value (v), which refers to the value of the action that agent takes from state to another. Furthermore, there are three major agent components, as mentioned in [30] which are *policy*, *value function*, and the *model*. *Policy* (π) refers to the agent behavior in the environment and which strategy is used to reach the goal, whether it is stochastic or deterministic policy. The *value function* (q) refers to the value of each state that has been reached by the agent to maximize the reward and to evaluate the effectiveness of the states. Finally, the *model* refers to the prediction algorithm or techniques that attempt to predict the next state based on the next immediate reward. To ensure consistency throughout the review paper, we primarily follow the notation established by [6]. The following subsection thoroughly explores RL and its algorithm categories, as shown in Figure 8. RL algorithms have been divided into two categories, model-based and model-free algorithms, which will be explained in detail in Section 4.3.1. Model-free algorithms are also divided into two parts, value-based and policy-based algorithms, which will be clarified in Section 4.3.2. Additionally, value-based algorithms are divided into two phases, on-policy and off-policy algorithms, as demonstrated in Section 4.3.3. Moreover, a comprehensive review of RL algorithms mentioned in Figure 8 is conducted in Section 4.4.

### 4.2. Markov Decision Process (MDP)

The MDP is recognized by various names, including “sequential stochastic optimization, discrete-time stochastic control, and stochastic dynamical programming” [31]. For the purpose of reinforcement learning, the MDP represents a discrete-time stochastic control mechanism that can be utilized to make decisions without the requirement of prior knowledge of the problem’s history, as in Markov Property [6,32]. Consequently, most reinforcement learning problems can be formalized as an MDP and can be solved with discrete actions. In other words, the MDP is a mathematical framework for modeling decision-making situations in which the outcome of a decision is uncertain. The MDP is similar to the Markov Reward Process but involves making decisions or taking actions [33]. The formal definition of the MDP is a five-tuple of (S, A, p, R, γ) [34], where:

S is a set of finite states that includes the environment.

A is the set of finite actions that an agent takes to go through all the states.

$p\left(s,\;a,\;s^{\prime }\right)$ is the transition probability matrix; it represents the trajectory of the agent ending up in state $s^{\prime }$ after taking an action a.

$R\left(s,\;a,\;s^{\prime }\right)$ is the reward function, which calculates the immediate reward after a transition from state s to $s^{\prime }$.

p and R are slightly different with respect to actions, as shown in Equations (1) and (2).

(1) $$p_{ss^{\prime }}^{a}=P\left[S_{t+1}=s^{\prime }|S_{t}=s,A_{t}=a\right]$$

(2) $$R_{s}^{a}=E\left[R_{t+1}|S_{t}=s,A_{t}=a\right]$$

γ is the discount factor, determining the significance of both of the immediate and future returns, where a discount factor γ∈[0, 1].

At each step (t), the learning agent observes a state s from S, selects an action a from A based on a policy π with parameters θ, and with probability. $p(s^{\prime }|s,a)$, moves to the next state $s^{\prime }$, receiving a reward r(s, a) from the environment.

**Figure 9:**
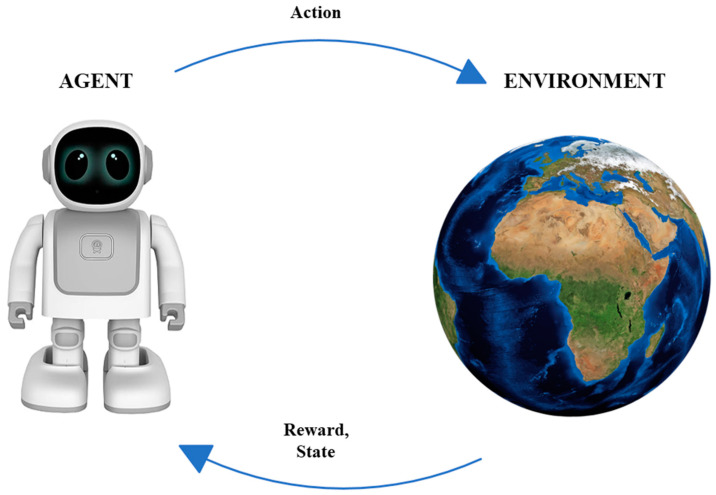
The MDP framework for modeling the interaction between an agent and its environment [6].

In essence, the MDP operates as follows: the agent takes an action a from the current state s, transitioning to another state $s^{\prime }$, guided by the transition probability matrix p. This iterative process persists until the agent reaches the final state with the highest possible reward, as depicted in Figure 8. These procedures are contingent on the value function of the state and the action, respectively. Through the value function, a policy function is derived to guide the agent in selecting the best action that maximizes the cumulative reward in the long run (Figure 9).

There are three different versions of Markov Decision Processes, which are used to model decision-making situations with different characteristics. These versions include fully observable MDPs (FOMDPs), partially observable MDPs (POMDPs), and semi-observable MDPs (SOMDPs) [25,35]. Fully observable MDPs (FOMDPs) refer to MDPs in which the agent possesses complete knowledge of the current state of the environment. Conversely, partially observable MDPs (POMDPs) involve scenarios where the agent lacks complete knowledge of the current state. In other words, the agent can only observe a portion of the environment’s state at each time step and must use this limited information for decision-making. Semi-observable MDPs (SOMDPs) are a variation of POMDPs in which the agent has some knowledge of the environment’s state, but this knowledge is incomplete and may be uncertain. In the following subsubsections, we will cover all the materials related to solving MDPs.

#### 4.2.1. Value and Policy Functions

Value functions are pivotal in all reinforcement learning algorithms as they estimate the future reward that can be expected from a given state and action [36,37]. Specifically, they measure the effectiveness of being in a specific state and taking a specific action, in terms of expected future reward, also known as expected return. To better understand the types of value and policy functions, it is essential to define the concept of return (denoted as $G_{t}$).

The return $G_{t}$ represents the cumulative reward that the agent receives through its interactions with the environment, as depicted in Equation (3) [38]. It is calculated as the sum of discounted rewards from time step t. The use of a discount factor is crucial to prevent the reward from becoming infinite in tasks that are continuous in nature. The agent’s objective is to maximize the expected discounted return, which balances the importance of immediate rewards versus future rewards, as determined by the discount factor.

(3) $$\begin{array}{c} G_{t}=R_{t+1}+\gamma R_{t+2}+\gamma^{2}R_{t+3}+\cdots =\overset{\infty }{\underset{k=0}{\sum }}\gamma^{k}R_{t+k+1} \end{array}$$

Interacting with the environment requires updating the agent’s value function (V(s)) or action-value function (Q(s, a)) under a specific policy [39]. The policy, represented as π(S)→A, is a mapping between states and actions that guides the agent’s decisions towards achieving the maximum long-term reward [38]. The policy determines the behavior of the agent and can be stationary, meaning that it remains constant over time. Mathematically, a policy can be defined in Equation (4) as follows:

(4) $$\begin{array}{c} \pi \left(a|s\right)=p\left[A_{t}=a|S_{t}=s\right] \end{array}$$

In reinforcement learning, the policy may manifest as deterministic or stochastic. A deterministic policy always maps a state to a specific action, utilizing the exploitation strategy. In contrast, a stochastic policy assigns different probabilities to different actions for a given state, promoting the exploration strategy.

According to aforementioned the policy above, the value function can be partitioned into two parts: the state-value function (v) and the action-value function (Q) [40]. The state-value function $v_{\pi }\;\left(s\right)$ represents the expected return for an agent starting in state s and then acting according to policy π. $v_{\pi }\;\left(s\right)$ is determined by summing the expected rewards at future time steps, with a given discount factor applied to each reward. This function helps the agent evaluate the potential value of being in a particular state as shown in Equation (5).

(5) $$v_{\pi }=E_{\pi }\;\left[G_{t}|S_{t}=s\right]=E_{\pi \;}\left[\overset{\infty }{\underset{k=0}{\sum }}\gamma^{k}R_{t+k+1}|S_{t}=s\right],\;\mathrm{for}\;\mathrm{all}\;s\in S\;$$

The action-value function $q_{\pi }\;\left(s,\;a\right)$ or Q-function represents the expected return for an agent starting in state s and taking action a, then operating based on policy π [40]. $q_{\pi }\;\left(s,\;a\right)$ is determined by summing the expected rewards for each state action pair as shown in Equation (6).

(6) $$q_{\pi }\left(s,\;a\right)=E_{\pi }\left[G_{t}|\;S_{t}=s,\;A_{t}=a\right]=E_{\pi }\left[\overset{\infty }{\underset{k=0}{\sum }}\gamma^{k}R_{t+k+1}|S_{t}=s,\;A_{t}=a\right]$$

By defining the principles of MDP for a specific environment, we may apply the Bellman equations to identify the optimal policy, as exemplified in Equation (7). These equations, developed by Richard Bellman in the 1950s, are utilized in dynamic programming and decision-making problems. The Bellman equation and its generalization, the Bellman expectation equation, are utilized to solve optimization problems where the latter accommodates for probabilistic transitions between states.

(7) $$v\left(s\right)=E\left[R_{t+1}+\gamma v\left(S_{t+1}\right)|S_{t}=s\right]$$

The state-value function can be decomposed into the immediate reward $\left(R_{t+1}\right)$ at time t+1 and the discounted value of the successor state at time $t+1\;\left(v\left(S_{t+1}\right)\right)$ multiplied by the discount factor (γ). This can be written as shown in Equation (8):

(8) $$v_{\pi }\left(s\right)=E_{\pi }\left[R_{t+1}+\gamma v_{\pi }\left(S_{t+1}\right)|S_{t}=s\right]$$

Similarly, the action-value function can be decomposed into the immediate reward $\left(R_{t+1}\right)$ at time t+1 on performing a certain action in the state s and the discounted value of the successor state at time $t+1\;\left(q\left(S_{t+1}\right)\right)$ multiplied by the discount factor (γ). This can be written as shown in Equation (9):

(9) $$q_{\pi }\left(s,\;a\right)=E_{\pi }\left[R_{t+1}+\gamma q_{\pi }\left(S_{t+1},\;A_{t+1}\right)|S_{t}=s,\;A_{t}=a\right]$$

After the decomposition of the state-value function and action-value function as described above, the optimal value functions can be obtained by finding the values that maximize the expected return. This can be carried out through iterative methods such as value iteration or policy iteration, which use the Bellman equations to update the value functions until convergence to the optimal values. Therefore, for a finite MDP, there is always one deterministic policy known as the optimal policy that surpasses or is equivalent to all other policies. The optimal policy leads to the optimal state-value function or the optimal action-value function. The optimal state value function is calculated as the highest value function v(s) across all stationary policies as shown in Equation (10):

(10) $$v_{*}\left(s\right)=\underset{\pi }{\max}v_{\pi }\left(s\right)$$

Likewise, the optimal action-value function is determined as the highest action-value function q(s, a) overall policies, as shown in Equation (11):

(11) $$q_{*}\left(s,a\right)=\underset{\pi }{\max}q_{\pi }\left(s,a\right)$$

#### 4.2.2. Episodic versus Continuing Tasks in RL

Reinforcement learning can be divided into two types of tasks: episodic and continuing. Episodic tasks are decomposed into separate episodes that have a defined endpoint or terminal state [41,42]. Each episode consists of a sequence of time steps starting from an initial state and ending at the terminal state, and a new episode begins. The objective of an episodic task is to maximize the total rewards obtained over a single episode.

In contrast, continuing tasks have no endpoint or terminal state, and the agent interacts with the environment continuously without any resets [41,42]. The continuing task aims to maximize the expected cumulative reward gained over an infinite time horizon.

### 4.3. Types of RL Models

This subsection introduces the differences between reinforcement learning models. Therefore, to delve deeper into reinforcement learning algorithms and their applications, it is important to understand the two categories they are divided into: model-free and model-based reinforcement learning algorithms. Additionally, there are two primary approaches in reinforcement learning for problem-solving, which are value-based and policy-based, both of which can be categorized under model-free methods [43]. Lastly, reinforcement learning algorithms can be categorized into two main types: on-policy and off-policy learning [6].

#### 4.3.1. Model-Based versus Model-Free RL Algorithms

Model-based reinforcement learning methods, also known as “Planning Model”, aim to learn an explicit model of the environment in which a complete and accurate understanding of how the environment works (a complete MDP), including the rules that govern the state transitions and the reward structure [36]. This understanding is typically represented as a mathematical model that describes the state transitions, the rewards, and the probabilities associated with each action. In other words, model-based reinforcement learning methods encompass the computation of action values through the simulation of action outcomes using a mental map or model of the environment that includes the environment’s various states, transition probabilities, and rewards [44,45]. The agent has the capability to acquire a model of the environment through experiential learning, enabling it to explore various trajectories of the map in order to choose the optimal action. The benefit of model-based learning is the ease with which the map can be modified to adapt to changes in the environment. However, this method is computationally expensive and time requirements, which may not be ideal for time-sensitive decisions. This model has several common algorithms, including model-based Monte Carlo and Monte Carlo Tree Search.

In contrast, model-free methods directly learn the optimal policy without explicitly modeling the environment’s dynamics, including the transition probabilities and the reward function [36]. In other words, model-free reinforcement learning is a decision-making approach where the value of various actions is learned through the process of trial-and-error interaction with the black box environment, without a world model [44,45]. Additionally, decisions are made based on cached values learned through the process of trial-and-error interactions with the environment. During each trial, the agent perceives the present state, takes an action relying on estimated values, and observes the resulting outcome and state transition. Subsequently, the agent calculates a reward prediction error, denoted as the disparity between the obtained outcome and the expected reward. This approach is more data-driven and is not contingent upon prior knowledge about the environment. The estimated values are used to guide action selection, and the values are updated trial-by-trial through a process of computing prediction errors. Once learning converges, action selection using model-free reinforcement learning is optimal. However, since the values rely on accumulated past experience, the method is less flexible in adapting to sudden changes in the environment, and it requires a significant amount of trial-and-error experience to become accurate. This model has several common algorithms including Q-learning, SARSA, and TD-learning that will be covered in the next section.

#### 4.3.2. Value-Based versus Policy-Based

A value-based method estimates the value of being in a specific state or action [46]. This method aims to find the optimal state-value function or action-value function from which the policy can be derived. For this reason, it is known as the indirect approach [47]. Value-based methods generally use an exploration strategy, such as ε-greedy or softmax, in order to guarantee an adequate exploration of the environment by the agent. Instances of value-based approaches encompass Q-learning and SARSA, which will be extensively discussed in the next section.

On the other hand, in a policy-based approach, the agent updates and optimizes the policy directly according to the feedback received from the environment, without the need for intermediate value functions [46]. This makes policy-based RL a conceptually simpler algorithm compared to value-based methods, as it avoids the computational complexities and approximations involved in estimating value functions [47]. Policy-based methods have demonstrated their effectiveness in learning stochastic policies that can operate in high-dimensional or continuous action spaces. This property makes them more practical than their deterministic counterparts, thereby widening their scope of application in real-world scenarios.

#### 4.3.3. On-Policy versus Off-Policy

An on-policy algorithm is based on a single policy, denoted as π, which is utilized by an agent to take actions in a given state s, aiming to obtain a reward [48]. In contrast, off-policy algorithms involve the use of two policies, the target policy and the behavior policy, denoted as π and μ, respectively [49,50]. The target policy is the one that the agent seeks to learn and optimize, while the behavior policy generates the observations that are used for learning. To ascertain the optimal policy, the agent uses the data generated by the behavior policy to estimate the value function for the target policy. Off-policy learning is a generalization of on-policy learning, as any off-policy algorithm can be converted into an on-policy algorithm by setting the target policy equal to the behavior policy.

### 4.4. RL Algorithms

This subsection presents the reinforcement learning algorithms along with their details. It focuses on three main algorithms: dynamic programming, Monte Carlo, and ends with temporal difference. The temporal difference algorithm is further divided into two methods: SARSA and Q-Learning.

#### 4.4.1. Dynamic Programming (DP)

Dynamic programming (DP) is a well-known model-based algorithm. DP consists of a collection of algorithms capable of determining the best policies if a complete model of the problem is available as MDP with its five-tuple of (S, A, p, R, γ) [6,25]. Additionally, DP is a general approach to solving optimization problems that involves breaking down a complex problem into smaller sub problems and solving them recursively. Dynamic programming represents a key concept that relies on value functions as a means to structure and organize the quest for optimal policies. Despite their ability to find optimal solutions, DP algorithms are not frequently used due to the significant computational cost involved in solving non-trivial problems [51]. Policy iteration and value iteration are two of the most commonly used DP methods. The optimal policies can be easily obtained through DP algorithms once the optimal value functions ($v^{*}$ or $q^{*}$) have been found, which satisfy the Bellman optimality equations as shown in Equations (12) and (13), respectively:

(12) $$v_{*}\left(s\right)=\underset{a}{\max}E\left[R_{t+1}+\gamma v_{*}\left(S_{t+1}\right)|\;S_{t}=s,\;A_{t}=a\right]$$

(13) $$q_{*}\left(s,\;a\right)=E\left[R_{t+1}+\gamma \;\underset{a^{\prime }}{\max}q_{*}\left(S_{t+1},\;a^{\prime }\right)|S_{t}=s,\;A_{t}=a\right]$$

**Policy iteration** is an algorithm in reinforcement learning that aims to find the optimal policy by iteratively improving a candidate policy through alternating between two steps: policy evaluation and policy improvement [52]. The goal of policy iteration is to maximize the cumulative returns, achieved by repeatedly updating the policy until the optimal policy is found. The process is called policy iteration because it iteratively improves the policy until convergence to an optimal solution is reached. The algorithm consists of two main parts: policy evaluation and policy improvement.

**Policy evaluation** is the process of estimating the state-value function $v_{\pi }$ for a given policy π [52]. This is often referred to as a prediction problem because it involves predicting the expected cumulative reward from a given state by following the policy π. The value function for all states is initialized to 0, and the Bellman expectation equation is applied to iteratively update the value function until convergence. This rule is utilized in Equation (14):

(14) $$v_{k+1}\left(s\right)=E\left[R_{t+1}+\gamma v_{k}\left(S_{t+1}\right)|S_{t}=s\right]\;\;\;\;\;\;\;\;\;\;\;\;\;\;\;\;\;\;\;\;\;=\underset{a}{\sum }\pi \left(a|s\right)\underset{s^{\prime },\;r}{\sum }p\left(s^{\prime },\;r|s,\;a\right)\left[r+\gamma v_{k}\left(s^{\prime }\right)\right]$$

**Figure 10:**
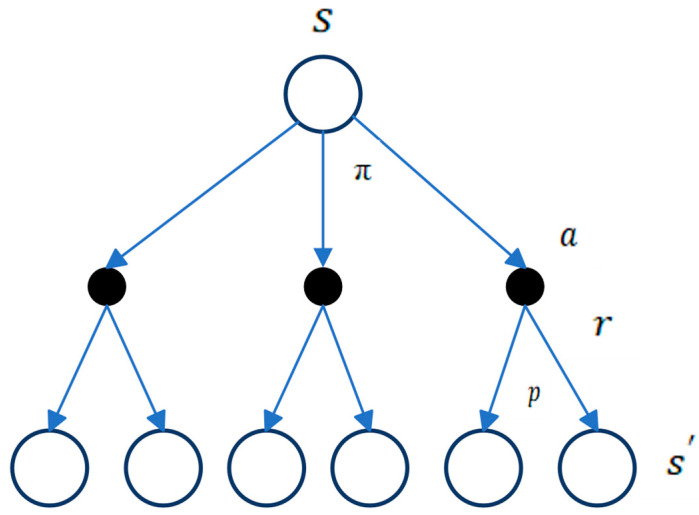
Backup diagram for $V_{\pi }$ [6].

The policy evaluation update rule involves k, which represents the $k^{th}$ update step, and $v_{k}+1$ represents the predicted value function under policy π after k+1 update steps, where $v_{k}=v_{\pi }$. This update, known as the Bellman backup, is depicted in Figure 10, illustrating the relationship between the value of the current state and the value of its successor states. In the diagram, open circles denote states, while solid circles represent state–action pairs. Through this diagram, the value information from successor states is transferred back to the current states. The Bellman backup involves iteratively updating the value function estimates for every state in the state space based on the Bellman equation until convergence is achieved for the given policy. This process is called iterative policy evaluation, and under certain conditions, it is assured to converge to the true value function $v_{\pi }$ as the number of iterations approaches infinity.

After computing the value function, the subsequent step is to enhance the policy by utilizing the value function. This step is known as policy improvement, and it is a fundamental stage in the policy iteration algorithm.

**Policy improvement** is a process in RL that aims to construct a new policy, which enhances the performance of an original policy, by making it greedy with respect to the value function of the original policy [52,53]. Policy improvement step seeks to improve the current policy by selecting the actions that lead to higher values $q_{\pi }\;\left(s,\;a\right)$ by considering the new greedy policy $\pi^{\prime }$, given by Equation (15).

(15) $$\pi^{\prime }\left(s\right)=\underset{a}{\mathrm{argmax}}q_{\pi }\left(s,\;a\right)$$

More precisely, during the policy improvement step, for each state in the state space, the action is selected that maximizes the expected value of the next state based on the provided value function. The resulting policy is guaranteed to possess a minimum level of quality equivalent to that of the original policy $\pi^{\prime }\geq \;\pi$ and may surpass it if the value function is accurate.

(16) $$v_{\pi^{\prime }}\left(s\right)\geq \;v_{\pi }\left(s\right)$$

**Figure 11:**
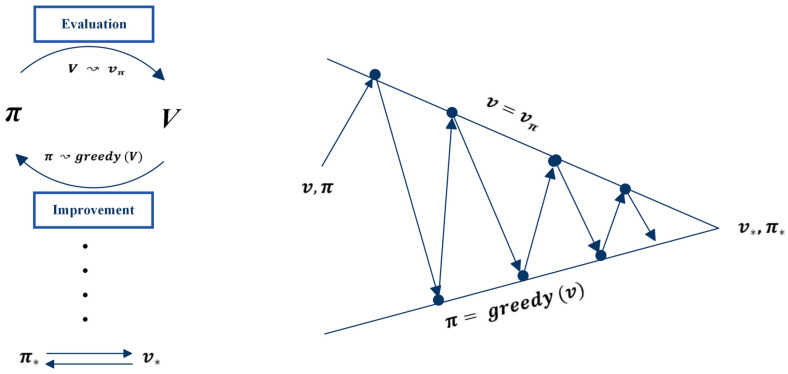
Illustrating the policy evaluation and improvement based on [6].

After improving a policy π using $v_{\pi }$ to generate a better policy $\pi^{\prime }$, the next step is to compute $v_{\pi^{\prime }}$ and use it to further improve the policy to $\pi^{\prime \prime }$. This process can be repeated to acquire a sequence of policies and value functions that improve monotonically, denoted as $\pi_{0},\;v_{\pi_{0}},\;\pi_{1},\;v_{\pi_{1}},\;\pi_{2},\;v_{\pi_{2}},\;\ldots .,\;\pi_{*},\;v_{*}$ as shown in Equation (17),

(17) $$\pi_{0}\overset{\;Ε\;}{\to }\;v_{\pi_{0}\;}\overset{\;1\;}{\to }\;\pi_{1}\;\overset{\;Ε\;}{\to }\;v_{\pi_{1}}\;\overset{\;1\;}{\to }\;\pi_{2}\overset{\;Ε\;}{\to }\ldots \;\overset{\;1\;}{\to }\;\pi_{*}\overset{\;Ε\;}{\to }\;v_{*}\;$$

until convergence to the optimal policy and optimal value function is achieved, where $v_{\pi^{*}}\left(s\right)\geq \;v_{\pi \;\neq \;\pi^{*}}\left(s\right)$ for all s∈S is found. For greater clarity on the policy iteration algorithm, Figure 11 illustrates the two components of this algorithm.

Value iteration commences by employing an initial arbitrary value function, subsequently proceeding to iteratively update its estimate to obtain an improved state value or action value function, ultimately resulting in the computation of the optimal policy and its corresponding value [6,25,37]. It is important that value iteration is a special case of policy evaluation in which the evaluation process terminates after one iteration. Furthermore, this algorithm can be derived by transforming the Bellman optimality equation into an update rule as shown below in Equations (18) and (19), respectively.

(18) $$v_{k+1}\left(s\right)=\underset{a}{\max}E\left[R_{t+1}+\gamma v_{k}\left(S_{t+1}\right)|S_{t}=s,\;A_{t}=a\right]$$

(19) $$q_{k+1}\left(s,a\right)=E\left[R_{t+1}+\gamma \underset{A_{t+1}}{\max}q_{k}\left(S_{t+1},A_{t+1}\right)|S_{t}=s,\;A_{t}=a\right]$$

As illustrated above, value iteration update involves taking the maximum over all actions, distinguishing it from policy evaluation. An alternative method to illustrate the interrelation of these algorithms is through the backup operation diagram, as shown in Figure 7, which is used to calculate $v_{\pi },\;v^{*}$. After obtaining the value functions, the optimal policy can be derived by selecting the actions with the highest values while traversing through all states. Similar to policy evaluation, this algorithm necessitates an infinite number of iterations to converge to $v^{*}$. It is important to note that these algorithms achieve convergence towards an optimal policy for a discounted finite MDP. Both policy and value iteration use bootstrapping, which involves using the estimated value of a future state or action to update the value of the current state $v_{k}\left(S_{t+1}\right)$ or action $q_{k}\left(S_{t+1}\right)$ during the iterative process. Bootstrapping offers the advantage of improving data efficiency and enabling updates that explicitly account for long-term trajectory information. However, a potential disadvantage is that the method is biased towards the starting values of $Q\left(s^{\prime },a^{\prime }\right)$ or $v\left(s^{\prime }\right)$.

#### 4.4.2. Monte Carlo (MC)

Unlike dynamic programming, where the model is completely known and used to solve MDP problems, Monte Carlo, also known as a model free algorithm, works with an unknown model of the environment, where the transition probabilities are unknown [6]. In MC, to gain experience, the agent must interact with the environment, which is then utilized to estimate the action value function. MC methods do not require prior knowledge of the environment’s dynamics to obtain optimal behavior; instead, they obtain experience–sample sequences of states, actions, and rewards [54]. Therefore, MC methods are utilized to find solutions to reinforcement learning problems based on average sample returns, which are updated after each trajectory. To ensure that returns are obtainable, MC methods exclusively utilize episodic tasks. In these tasks, the agent interacts with the environment for a fixed number of time steps; the episode terminates after a specific goal is achieved or a terminal state is reached. Moreover, only complete episodes can estimate the values and change the policies, which means that they are incremental in an episode-by-episode sense.

In MC methods, the return of an action in one state is estimated by sampling and averaging returns for each state–action pair [55]. However, since the action selections are learned and updated in each episode, the problem is considered nonstationary, as the return of an action in one state is determined by the actions taken in subsequent states within the same episode. To overcome this nonstationary situation, a General Policy Iteration (GPI) approach is used. In GPI, value functions are learned from sample returns using MC methods rather than computing them from knowledge of the MDP as in dynamic programming.

To determine $v_{\pi }$, the general idea of MC methods is to estimate it from experience by averaging the returns observed after visiting each state. The more returns observed, the closer we can become to the expected value. There are various approaches to estimate $v_{\pi }\left(s\right)$, which is the value of a state s under a prescribed policy π. This estimation is achieved by using a collection of episodes obtained by following π and traversing through s. In each episode, the state s may be visited more than once. Therefore, there are different approaches for estimating and updating $v_{\pi }\left(s\right)$, which are as follows:

1. First-Visit MC Method
  This method has been extensively studied since the 1940s [6]. This approach considers only the first visit of each state in each episode when computing the average return for that state [54]. The First-Visit MC Method can provide an estimate of the true state-value function by averaging the returns acquired on each first visit. As the number of first visits to states approaches infinity, this method converges to the optimal state-value function.
2. Every-Visit MC Method
  The Every-Visit MC approach exhibits dissimilarities when compared to the First-Visit MC method in that it averages the returns received after every visit to a state across all episodes, rather than just the first visit [56]. The value function estimate for a state is updated after every visit to the state in an episode, regardless of whether it has been visited before. Similar to the First-Visit Method, the Every-Visit Method converges to the optimal state-value function as the number of visits to each state approaches infinity

**Figure 12:**
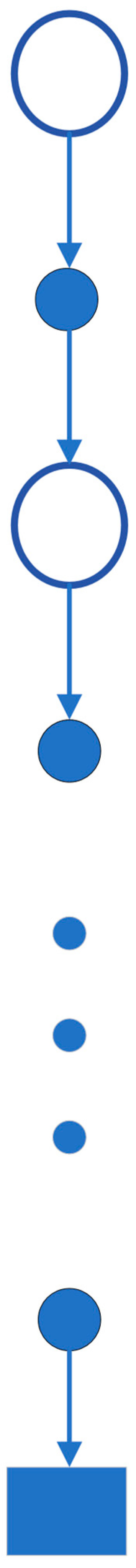
Monte Carlo Backup Diagram [6].

Similar to dynamic programming, the Monte Carlo algorithm employs a backup diagram, as shown in Figure 12; however, it differs from the one used in DP. In the MC diagram for estimating vπ, a state node is located at the root, representing the initial state of the episode. The diagram demonstrates the sequence of transitions that take place during a single episode and ends at the terminal state, marking the conclusion of the episode. The MC diagram extends to the end of the episode since Monte Carlo methods necessitate complete episodes to estimate values and update policies based on average returns.

In cases where the environment is unknown, Monte Carlo methods offer a suitable approach for estimating the value of state–action pairs, as opposed to state values. This is due to the fact that state–action pair estimation provides more informative measures for determining the policy [57]. The policy evaluation problem is utilized for the action-value $q_{\pi }\left(s,\;a\right)$ to estimate $q_{*}$ in Monte Carlo, which represents the expected return when starting from state s, taking action a, and then following policy π. There are two approaches for estimating state–action values in MC: First-Visit and Every-Visit approaches. The First-Visit MC Method computes the average of returns following the initial visit to each state and the action pair within an episode. Conversely, the Every-Visit MC Method estimates the value of a state–action pair by averaging the returns from all the visits to it. These two approaches converge as the number of visits to a state–action pair approaches infinity.

The main problem with MC methods is that numerous state–action pairs may remain unvisited if the policy is deterministic. To address this issue, the exploring starts assumption is utilized. This assumption dictates that episodes begin from a state–action pair, with each pair having a non-zero probability of being chosen as the starting point. This ensures that every state–action pair will be visited an indefinite number of times as the number of episodes’ approaches infinity.

The MC control algorithm uses the same concept of Generalized Policy Iteration as in DP. To obtain an optimal policy, classical policy iteration is performed by starting with an arbitrary policy π_0 and iteratively conducting policy evaluation and improvement until convergence, as shown below.

(20) $$\begin{array}{c} \pi_{0}\overset{\;Ε\;}{\to }\;q_{\pi_{0}\;}\overset{\;1\;}{\to }\;\pi_{1}\;\overset{\;Ε\;}{\to }\;q_{\pi_{1}}\;\overset{\;1\;}{\to }\;\pi_{2}\overset{\;Ε\;}{\to }\ldots \;\overset{\;1\;}{\to }\;\pi_{*}\overset{\;Ε\;}{\to }\;q_{*}\; \end{array}$$

where $\overset{E}{\to }$ means a complete policy evaluation and $\overset{I}{\to }$ means a complete policy improvement. Policy evaluation is conducted using the same method as in DP. Policy improvement is achieved by adopting a policy that follows a greedy approach concerning the current value function. The optimal policy can be extracted by selecting the action that maximizes the action-value function.

(21) $$\begin{array}{c} \pi \left(s\right)=\underset{a}{\mathrm{argmax}}q_{\pi }\left(s,\;a\right) \end{array}$$

In Monte Carlo policy iteration, it is customary to alternate between policy evaluation and policy improvement on an episode-by-episode basis. Once an episode is completed, the observed returns are utilized to evaluate the policy. Subsequently, the policy is enhanced with every state visited during the episode. For a detailed description of on-policy and off-policy MC algorithms, refer to [6].

There are several key differences between Monte Carlo (MC) and dynamic programming (DP). For example, MC estimates are based on independent samples from each state, while DP estimates rely on estimating values for all states simultaneously, taking into account their interdependencies. Another key difference is that MC is not subject to the bootstrapping problem because it uses complete episodes to estimate values. In contrast, DP estimates rely on one-step transitions. Furthermore, MC estimates state–action values by averaging the returns obtained from following a policy until the end of an episode, whereas DP focuses on one-step transitions. Finally, MC learns from experience that can be obtained from actual or simulated episodes. These differences between DP and MC show that the temporal difference (TD) learning algorithm was developed to overcome the limitations of both DP and MC techniques by combining ideas from both approaches. Its main goal is to provide a more efficient and effective approach to reinforcement learning which will be discussed in the next section.

#### 4.4.3. Temporal Difference (TD)

Temporal difference learning is a model-free RL model and it is widely regarded as a fundamental and pioneering idea in reinforcement learning [6,56]. As mentioned in the previous section, temporal difference learning is a combination of the ideas of both Monte Carlo and dynamic programming. Therefore, the TD algorithm learns from experience where there is an unknown model or no model of the environment’s dynamic, similar to MC [58]. On the other way, TD, like DP algorithms, updates estimates depending on the other learned estimates without waiting for a whole episode to be finished. Therefore, the TD method bootstraps like DP too. There are two problems to discuss with this algorithm: prediction and control problems [54]. The prediction problem regards estimating the value function $\nu_{\pi }$ for a given policy π. For the control problem, TD, like MC and DP methods, uses the idea of General Policy Iteration (GPI) to find the optimal policy.

TD methods update their value function at each time-step t+1 by incorporating the observed reward $R_{t+1}$ and the estimated value $V\left(S_{t+1}\right)$. The value and action-value function updates for TD methods can be expressed using the following equation:

(22) $$V\left(S_{t}\right)\leftarrow V\left(S_{t}\right)+\alpha \;\left[R_{t+1}+\gamma V\left(S_{t+1}\right)-V\left(S_{t}\right)\right]$$

**Figure 13:**
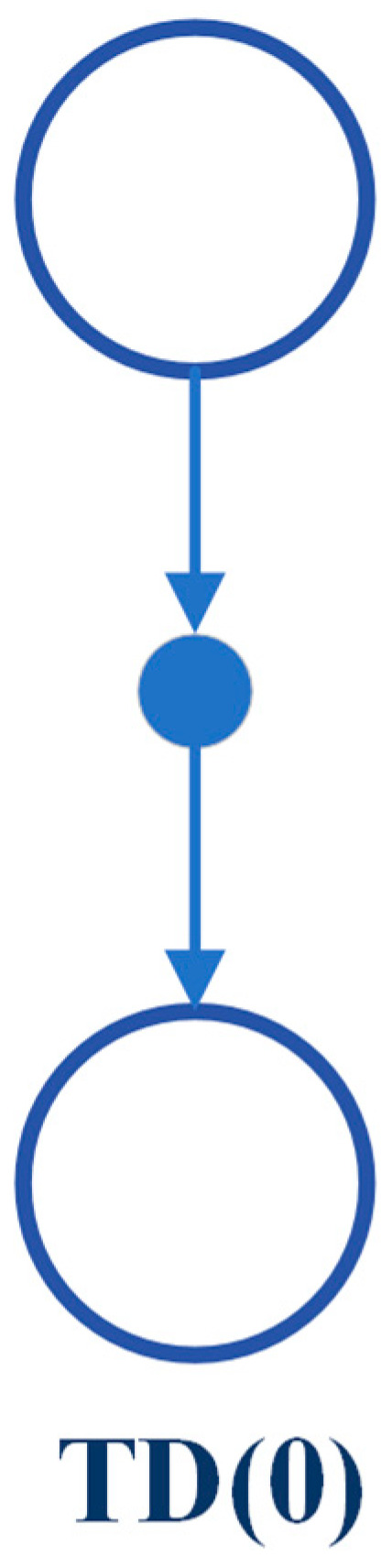
TD Backup Diagram [6].

Here, ← refers to the update operator, α is a constant step-size parameter, and γ is the discount factor. This particular method is known as TD (0) or one-step TD. The backup diagram for TD (0) shows that the value estimates for the state node positioned at the summit of the diagram are updated based on one sample transition from the current state to the subsequent state, as shown in Figure 13.

TD (0) update can be understood as an error that quantifies the disparity between the estimated value of $S_{t}$ and the better estimate $R_{t+1}+\gamma V\left(S_{t+1}\right)$. This error is known as the TD error and is represented by the following equation:

(23) $$\delta_{t}=R_{t+1}+\gamma V\left(S_{t+1}\right)-V\left(S_{t}\right)$$

where $\delta_{t}$ represents the TD error at time t. As the agent traverses through each state–action pair multiple times, the estimated values converge to the true values, and the optimal policy can be extracted using Equation (21).

(24) $$\pi \left(s\right)=\underset{a}{\mathrm{argmax}}q_{\pi }\left(s,\;a\right)$$

##### SARSA

**Figure 14:**
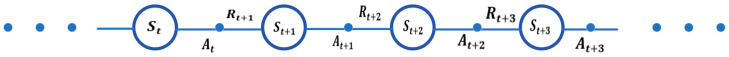
SARSA algorithm [6].

SARSA is an on-policy TD control algorithm where the behavior policy is exactly the same as its target policy [59]. This method must estimate $q_{\pi }\left(s,\;a\right)$ for the current behavior policy π and for all the states s and actions a by using the same TD method as previously explained and shown in Figure 14.

Therefore, this algorithm considers transitions from a state–action pair to a state–action pair, learning the values of the state–action pair. As SARSA is an on-policy approach, the update of the action-value functions is performed using the equation below.

(25) $$Q\left(S_{t},\;A_{t}\right)\leftarrow Q\left(S_{t},\;A_{t}\right)+\alpha \;\left[R_{t+1}+\gamma Q\left(S_{t+1},\;A_{t+1}\right)-Q\left(S_{t},\;A_{t}\right)\right]$$

The update is performed after each transition from a non-terminal state $S_{t}$; when the $S_{t+1}$ is terminal, the value of $Q\left(S_{t+1},\;A_{t+1}\right)$ is set to 0. This algorithm uses all the elements of the quintuple $\left(S_{t},\;A_{t},\;R_{t+1},\;S_{t+1},A_{t+1}\right)$ that takes a transition from one state–action pair to another, leading to the naming of the algorithm as SARSA, which stands for state–action–reward–state–action. The estimation of $q_{\pi }$ continues for the behavior policy π, and the policy changes to the optimality with respect to $q_{\pi }$. The SARSA algorithm converges to an optimal policy and action-value function by using ε-greedy or ε-soft with the probability of 1, under the condition that all state–action pairs are visited infinitely.

##### Q-Learning

Q-learning is a widely recognized off-policy algorithm in reinforcement learning (RL). The key feature of Q-learning is that it estimates the action-value function Q which leads to directly approximating $q_{*}$ (the optimal action-value function), regardless of the policy being executed [58,59]. This technique is defined in Equation (24) as follows:

(26) $$Q\left(S_{t},\;A_{t}\right)\leftarrow Q\left(S_{t},\;A_{t}\right)+\alpha \;\left[R_{t+1}+\gamma \underset{a}{\max}Q\left(S_{t+1},\;a\right)-Q\left(S_{t},\;A_{t}\right)\right]$$

where the Q-learning updates use only the four elements $\left(S_{t},A_{t},R_{t+1},S_{t+1}\right)$ while assuming $S_{t+1}$ is a decision variable to optimize the action-value function. This approach guarantees that the agents can determine the optimal policy based on the assumption that each state–action pair is visited an infinite number of times. It has been demonstrated that Q converges to a particular value with probability 1 to $q_{*}$.

### 4.5. Comparison between DP, MC, and TD

A brief comparison between the DP and MC algorithms has been mentioned at the end of the MC algorithm subsection. However, a comprehensive comparison of dynamic programming (DP), Monte Carlo (MC), and temporal difference (TD) reinforcement learning (RL) algorithms is presented in Table 3. Table 3 summarizes the characteristics of each algorithm, including their requirement for a model of the environment to learn value functions, which is only necessary for DP. Both MC and TD algorithms learn value functions from sampled experience sequences of states, actions, and rewards. MC does not suffer from the bootstrapping problem because it uses complete episodes to estimate value functions, whereas DP and TD use bootstrapping to estimate value functions because they rely on the previously estimated value functions. This leads to unwanted bias in the estimates. In contrast, MC algorithm estimates are based on independent samples from each state, which avoids the estimation bias. However, this method introduces high variance because the estimate of a value function is proportional to the variance of the returns. Since the returns from different episodes can have high variance because of the stochastic nature of the environment and the policy, the estimate of the value function can have high variance as well. In terms of on-policy or off-policy, DP and MC algorithms are on-policy methods, whereas TD is on-policy and off-policy method. In terms of computational cost, DP requires simultaneous updates of all value functions, making it computationally expensive. MC methods update value functions at the end of each episode, whereas TD updates them after a one-time step. Generally, model-based algorithms like DP converge faster than model-free algorithms like MC and TD. However, among model-free algorithms, TD converges faster than MC as it does not wait for only one time step to update value functions.

### 4.6. Function Approximation Methods

Since we have discussed traditional RL algorithms and their role in solving MDP problems, it is important to note that MDPs typically involve discrete tasks where states and actions can be represented as arrays or tables, manageable by value functions. In fundamental RL algorithms, value iteration assigns values to states, facilitating the discovery of optimal value functions and policies. However, in complex environments with large state spaces, this approach becomes impractical due to high computational costs.

To address this challenge, the adoption of function approximation methods becomes imperative. These methods generalize value functions through parameterized functional structures instead of relying on tables [6,25]. Rather than storing values for each state separately, function approximation methods represent states using features and weights. A common form of approximate value function is expressed as follows:

(27) $$\hat{v}\;\left(s,\;w\right)\approx \;v_{\pi }\left(s\right)$$

where $\hat{v}\;\left(s,\;w\right)$ represents the approximated value function for state s, and $w\;\in \;R^{d}$ denotes the weight vector parameter. $v_{\pi }\left(s\right)$ denotes the true value function under policy π for state s. The parameters w undergo adjustments throughout the training process in order to reduce the difference between the approximated and true value functions. These adjustments can be carried out by utilizing methods such as gradient descent (SG) or stochastic gradient descent (SGD). The benefits of function approximation include scalability, generalization, and sample efficiency. There are various types of function approximation, such as linear functions, Fourier basis functions, and non-linear neural network function approximation. To delve deeper into function approximation and its types, readers are encouraged to consult references [6,25]. In the next subsection, we will explain the rise of deep reinforcement learning.

#### The Combination of (Deep Learning and Reinforcement Learning)

Deep learning and reinforcement learning are two powerful techniques in AI [6,23]. Deep learning employs a layered architecture that enables automated feature extraction, eliminating the need for manual feature engineering processes. Additionally, deep learning techniques excel in handling high-dimensional data due to their capability of automating feature extraction. Therefore, the combination of deep learning and reinforcement learning leads to deep reinforcement learning (DRL), where DRL addresses problems in which MDP states are high-dimensional and cannot be effectively solved by traditional RL algorithms. In DRL, deep neural networks are implemented for function approximation in Q-learning [25]. Table 4 illustrates the emergence of deep reinforcement learning algorithms.

## 5. RL Application

This section presents a literature review of the most influential research papers on the application of reinforcement learning (RL) in both robotics and healthcare systems. A comparative analysis is provided between the articles, including the techniques employed and their corresponding outcomes. In the context of robotics, the focus is primarily on the use of RL algorithms for object grasping and manipulation, a rapidly developing research area with promising potential. Additionally, for healthcare applications, the emphasis is on the use of RL methods for addressing cell growth problems, an area of increasing interest due to its significance in optimizing cell culture conditions, drug discovery, and enhancing understanding of cellular behavior, among other potential benefits.

### 5.1. Robotics

Humans have a direct sensorimotor connection to the environment, enabling them to see and observe objects and determine how to pick them up. However, robots have lagged far behind in possessing these capabilities [65]. Even tasks that are considered trivial for humans can pose significant challenges for robots. Robotic grasping and manipulating objects in unstructured and dynamic environments remain critical and challenging problems due to the variability and complexity of the real world. Traditional machine learning (ML) approaches often struggle to handle the diversity of objects in terms of size, weight, texture, transparency, and fragility [12]. Moreover, dealing with cluttered scenes and managing uncertainties in perception and control proves even more challenging for ML. Consequently, reinforcement learning (RL) has emerged as a solution, allowing robots to learn through trial and error and adapt to various situations [66]. RL has gained significant traction in robotics, particularly in the field of grasping and manipulation [67]. In this section, we review some of the most recent influential papers on RL-based robotics applications for grasping and manipulation as shown in Table 5 and Table 6, respectively. We analyze the methods and results of these studies and discuss the challenges and limitations they have encountered in this field. For clearer and higher-resolution framework figures used in Table 5, please refer to Supplementary Materials.

In [68], the authors introduce a graph-based Q-learning model for grasping/pushing occluded objects. This model consists of an encoder, a graph reasoning module, and a decoder. In the encoder phase, state features are merged to facilitate the incorporation of the features of one region to contain features from other regions, leading to improved feature learning. Next, graph reasoning is used to integrate the features of adjacent regions. Finally, in the decoder phase, the updated features are mapped to specific features. The authors employ DQN as the underlying algorithm and design a graph Q-Net to predict the Q-value. The experiment consists of two tasks: exploration and coordination. In the exploration phase, the robot only employs push actions, while in the coordination phase, the robot requires both grasping and pushing actions for cooperation. The experiment was conducted in two environments using a simulation with a UR5 robot arm and an RG2 gripper in the V-REP simulation environment, as well as with a real robot. To evaluate this model, two metrics were used: the rate of success (RS) and the motion number (MN). The results of the proposed model, using a dataset of 10 scenes with block shapes, achieved an RS of 100% and an average MN of less than 2 in the exploration task. In the coordination task, the model achieved an RS of 91% with an average MN of 3.2. When the dataset was extended to include 20 different scenes, in coordination tasks, the model achieved an SR of 95% with an average MN of 2. On the other hand, the experiment with a real robot achieved an SR of 91% with an average MN of 7.3. These results surpass the state-of-the-art results of previous methods.

In [69], the authors introduce a VAGS strategy based on DRL that aims to empower the robot to independently adjust the camera viewpoint in achieving swift and precise grasping in cluttered scenes. Through experiments, the authors demonstrate that the VAGS method improves the grasping success rate (GSR), scene clearing rate (SCR), and grasping efficiency (GE) in both simulation and real-world scenarios. Simultaneously, they suggest a DAES method based on ε-greedy to expedite the training of VAGS and introduce a reward function to tackle the issue of sparse reward in RL. The model’s generalization ability is enhanced by randomly generating variations in color, shape, quantity, and posture of objects throughout the training phase. In the simulation experiment, when applying VAGS to scenes with 1 to 10 objects, the GSR was 83.50%, and the SCR was 95%. Subsequently, DAES was applied to VAGS to enhance training efficiency. The results, with and without VANet (viewpoint adjusting), showed an average GSR improvement (w/o VANet = 76.38%, w/VANet = 86.87%) and SCR improvement (w/o VANet = 84%, w/VANet = 95%). In real-world experiments, the results showed a GSR of 83.05%, an average grasping time of 8.5 s, a mean picks per hour of 348, and a motion number of 1.38. The aforementioned findings suggest that the suggested framework surpasses prior frameworks.

In [70], this article introduces a learning-based method that employs simulation data to instruct a robot in object manipulation. It employs two model-free RL algorithms. The first algorithm relies on the on-policy RL algorithm known as Proximal Policy Optimization (PPO) to train the controller of the robot. Additionally, the learning process incorporates an off-policy algorithm called Soft Actor-Critic (SAC) to assess learning performance. Furthermore, the article proposes a fine-tuning procedure that initializes the policy for the target task with the acquired policy for the base task, diminishing the necessary count of episodes and accelerating training. The objectives of this model encompass successful grasping and lifting of target objects, effective management of the robot’s redundancy to prevent exceeding joint constraints, evading obstacles to avoid collisions, and the refinement of control actions to facilitate the application of the acquired controller on an actual robot. The design of the reward function aims to guide the learning concerning task success/failure, the level of performance, and the consideration of safety. Consequently, experiments conducted in both simulation and real-world scenarios, using the Franka Emika Panda robot, have achieved a 100% success rate. This promising outcome suggests the potential applicability of the proposed approach in real-world settings.

In [71], the authors introduce an approach to robotic manipulation, employing Simulated Locomotion Demonstrations (SLDs) in conjunction with reinforcement learning (RL). This method is distinctive in that it does not necessitate human demonstrations, instead capitalizing on the notion that any robotic manipulation task can be interpreted as a form of locomotion task when viewed from the object’s perspective. By employing a practical physics simulator, an object locomotion policy is derived, which is subsequently utilized to produce supplementary rewards termed Simulated Locomotion Demonstration Rewards (SLDRs). The primary method used in this study is Deep Deterministic Policy Gradient (DDPG) in conjunction with sparse rewards. Additionally, the authors have incorporated the following algorithms as baselines alongside DDPG-Sparse, namely, HER-Sparse and HER-Dense. DDPG-Sparse + SLDR and HER-Sparse + SLDR denote the proposed approach, integrating SLDR with distinct methods for policy learning. The approach has been assessed across 13 tasks, achieving a commendable 100% success rate. The outcomes demonstrate its competitiveness with state-of-the-art approaches that depend on human demonstrations.

In [72], the authors discuss the application of Dueling Deep Q-Learning (DDGN) to acquire a grasping policy. The authors employ a five-DoF robotic arm to address the challenge of grasping a target object. This study not only explores the advantages of utilizing reinforcement learning algorithms for this purpose but also substantiates this strategy with continuous visual feedback. The DDGN algorithm is trained using a reward function specifically designed for it, utilizing visual data obtained from a Kinect camera through a simulation environment called the Webats Simulator. The presented approach represents an enhanced deep reinforcement learning algorithm structured for end-to-end learning, primarily reliant on vision-based robotic grasping. This architecture, with the assistance of a custom-designed CNN model, enables the agent to execute appropriate grasping actions. The results indicate that a minibatch size of 16 yields superior results in terms of immediate reward value when compared to a minibatch size of 32, utilizing an Epsilon-greedy exploration strategy.

In [73], the authors discuss a vision-based approach to robotic object grasping that utilizes DRL to enable robots to achieve a high success rate in grasping objects. The introduced technique combines computer vision and deep RL to facilitate the learning and improvement of the robots’ grasping capabilities. The YOLO algorithm is used to detect, localize, and recognize objects in images, reducing training time by providing object location as input to deep RL. Additionally, the Soft Actor-Critic deep RL algorithm, an off-policy framework, enhances sample efficiency. This article trains the robot manipulator by using SAC to adopt object grasping via self-learning. The experiments were conducted in both simulation and real-world environments. The authors utilized the V-REP robot simulator to train SAC, and the results demonstrated a reduction in total training time and grasping attempts when compared to the approach without it. Moreover, the authors successfully transferred the trained SAC to a real 6-DoF robot manipulator, which performed object grasping and pick-and-place actions effectively, even for previously unseen objects.

In [74], the authors present a new approach to robotic grasping using dual-agent deep reinforcement learning called QMIX-PSA. This approach consists of two agent networks, a PA-Net and a SA-Net, which are utilized to anticipate grasp position and orientation. QMIX estimated the joint action value of these two networks in order to link them. Then, the authors extended their four-DoF into a six-DoF one and attempted to eliminate disturbances using augmented rewards. This approach has several advantages that make it suitable for such a problem. An extensive experiment has been conducted using this approach and six of its peers across three different scenarios: single, scattered, and cluttered objects. The results showed that QMIX-PSA outperforms its peers in terms of grasp success rate and quality, especially in cluttered scenarios where existing SGL methods are less competent.

In [75], the authors presented a method called G-PAYN for the iCub humanoid robot based on DRL, which uses automatically gathered offline demos. The research proceeds by proposing a modular pipeline for grasping an object with the iCub humanoid, consisting of two stages. The first stage involves grasp pose computation, which is performed by external algorithms, specifically superquadric models and the grasp pose generator VGN. The second stage involves the execution of the grasp. Both stages are utilized to start the movement. Therefore, a control policy was presented for these two steps to create an automated approach for acquiring grasping demos and then learning the policy using the SAC algorithm and previously obtained data. The experimental phase was conducted in a simulation environment using the MuJoCo simulator with five objects from the YCB video dataset. To evaluate the proposed approach, G-PAYN, was compared to four different baselines, namely Demonstrations Pipeline, SAC, OERLD, and AWAC. The results demonstrated that G-PAYN excels in half of the experiments, achieving a high success rate while delivering an equivalent performance in other circumstances.

In [76], the authors introduce self-supervised deep reinforcement learning (DRL) for performing pick-and-place operations on objects of various shapes. In this framework, the agent learns how to perform a series of prehensile (grasping actions) and non-prehensile (left-right slides) robotic manipulations using a model-free and off-policy DRL, specifically Q-Learning. This learning process relies on trial and error. Notably, this approach facilitates bidirectional learning of sliding and pushing operations. The Deep Q-Network (DQN) consists of three fully convolutional networks (FCN) that use DenseNet-121′s memory-efficient architecture. Consequently, agents acquire knowledge and converge to optimal policies within an end-to-end memory-efficient framework utilizing pixel-wise parameterization. Actions with the highest Q-value are executed at the 3D location of a pixel expected to possess the maximum Q-value. The reward scheme is designed based on the successful outcomes of actions, including grasping, left and right slides, or taking no action. Finally, the proposed approach has been evaluated and compared with various baseline approaches in terms of success rate and grasping success.

In [77], the authors introduce a novel pipeline that combines traditional control and reinforcement learning (RL) techniques for both simulated and real-world environments to validate RL methods across various scenarios, including reach, grasp, and pick-and-place tasks. Two algorithms, Soft Actor-Critic (SAC) and Proximal Policy Optimization (PPO), are employed in this study. SAC, as the first method, is an off-policy RL algorithm that effectively balances the exploration–exploitation tradeoff and allows for ease of parallelization. Conversely, PPO is a policy gradient technique known for its ability to provide rapid policy updates. In the simulation environment, the authors selected the PyBullet Python-based environment for its adaptability and ease of modification. Given that hyperparameter tuning is often necessary for the chosen algorithms, Optuna is utilized as a hyperparameter optimization framework. Additionally, the authors employed two reward structures in this study: dense and sparse. For the real-world experiments, the Panda Research Robot was used. The utilization of the Franka control interface (FCI) in this robot enables the establishment of bidirectional communication between the agent and the workstation. This communication facilitates the exchange of positional readings and commands. In the Panda robot, Franka-ROS and MoveIt are the control packages employed to test connections between the simulated PyBullet agent and the real-world robot. The results obtained in the simulated environment indicate that PPO performed better in complex tasks, while SAC excelled in simpler tasks. However, in real-world scenarios, there was a reduction in performance of approximately 10–20% across all tasks when compared to the simulation environment due to the geometry difference between the objects in both environments. Table 6 displays the outcomes and limitations of each article, providing a general overview of research conducted in the realm of robotic grasping and manipulation.

### 5.2. Healthcare

In recent years, healthcare applications have garnered considerable attention, owing to the substantial integration of artificial intelligence (AI) techniques, notably reinforcement learning (RL) [14,78]. As previously mentioned, RL is a subfield of machine learning that revolves around decision-making and control in dynamic environments. The growing interest in RL can be attributed to its capacity to optimize intricate processes and adapt to changing conditions within the healthcare sector. Consequently, RL has proven to be effective in addressing a variety of healthcare challenges [15,79,80,81,82]. Among the most recent and promising areas in healthcare is the resolution of cell growth problems, which hold critical significance in diverse healthcare applications like tissue engineering, regenerative medicine, and cancer treatment [79,83]. This review, therefore, concentrates on the topic of cell growth problems and explores the application of RL by scientists to solve such challenges.

As may be known, all living organisms are composed of one or more cells, which serve as the building blocks of life [84]. Cells are the basic units of life that can perform all vital functions of an organism, such as metabolism, growth, and reproduction. They come in various shapes and sizes and can be single-celled or found in multicellular organisms. For the purposes of this review, two different types of cells were utilized. Yeast cells and mammalian cells are two distinct types of cells. Yeast cells are single-celled, eukaryotic organisms that are commonly used in fermentation processes such as brewing beer, baking, and biofuel production. On the other hand, mammalian cells are multicellular eukaryotic cells that form the tissues and organs of mammals. There are indeed several problems related to yeast cells and mammalian cells in terms of biotechnology, such as controlling the gene expression of the cells and the tolerance of yeast cells to different environmental conditions such as high temperature, as well as optimizing the conditions of culture for mammalian cells, such as nutrient concentrations, temperature, and other factors to achieve optimal growth rate. Therefore, this literature review will explore the current understanding and research on cells, with an emphasis on using machine learning approaches to identify and solve problems related to different types of cells. The topics covered will include the structure and function of cells, as well as recent advancements and discoveries in the field of biotechnology. This review will also examine challenges and questions that remain to be addressed in the field of biotechnology, specifically related to the application of machine learning to cells.

The authors of [85] describe a new approach for collecting accurate data on cells. The authors present an optimization of cell cycle measurement through the development of a multicell time-lapse imaging system. The system includes an integrated, motorized inverse microscope that is housed within a CO_2_ incubator to maintain optimal growth conditions. It also features CCD cameras for imaging, specialized illumination, steering electronics, a computer, and a control system. This protocol is distinct from previous methods in that it does not impose a time limit on cell growth, enabling uninterrupted observation and collection of data. The goal of this protocol is to measure the length of the cell cycle of individual HaCat cells using long-term scanning microscopy. The length of the prokaryotic cell cycle varies depending on the complexity of the organism. To understand the processes involved in the mammalian cell cycle, the authors provide a detailed description of cell cycle division and its subphases. This includes the division of the nucleus (mitosis) and the separation of the two daughter cells (cytokinesis). Mitosis is the first part of cell cycle division, in which the cell divides into two new nuclei with replicated chromosomes, while cytokinesis is the process of physically dividing the cell into two daughter cells. Additionally, the first cell cycle includes the phases G1, S, G2, where G1 and G2 are the variables of the division and S and M are the constants of the cycle. Understanding the duration of the cell cycle has implications for mass protein production in the food industry, reducing cell growth to aid in cancer treatment, and determining the cell cycle length. In this protocol, the authors aim to determine the cell cycle length of HaCat cells using a long-term scanning microscopy. They used two HaCat cell cultures from a frozen cell stock under similar conditions, starting the experiment at 4 pm in the afternoon. They attempted to measure the phase of the cell cycle length during the first cell cycle growth, with a duration of 17 h. They then used time-lapse microscopy, which can extend to 2 days and last for 2 weeks, based on cell division. They set up four congruent imaging systems inside a CO_2_ incubator, one for control and the others for cell treatments. They also installed suitable software to divide the computer screen into two parts to visualize both cell cultures, turned on autofocus for high-quality images, and adjusted image acquisition parameters for maximal gray-scale dynamic range resolution. Finally, they converted the images taken every minute into a video file by speeding up the exposures from 1 s to 30 s.

The authors of [86] introduce a new long-term scanning-perfusion platform that addresses the limitations of current methods in cell culture. The platform includes features such as replacing an old medium with a fresh medium, avoiding physical contact with the cells, providing uninterrupted imaging of single cells, and maintaining near-physiological conditions for several weeks. The system was validated using serum starvation and chemical induction of cell cycle arrest in HaCaT cells. The perfusion operation used in this platform aims to improve cell productivity in bioreactors, reduce waste of nutrients, and eliminate the side effects of high flow rate without removing cells from the bioreactors. The system includes time-lapse video microscopy, electronic steering, and a computer system. The perfusion subsystem is used to protect the cells during experiments and observe them for several generations without physical contact. The TL microscopy subsystem includes an inverse microscope, a high-sensitivity camera, specialized illumination, and a control unit. The system is controlled by the open-source software Fiji, which is used for quantitative image analysis.

The previous two articles introduced a new system for collecting data on cellular processes using time-lapse microscopy. However, this method can be quite labor-intensive, as the segmentation of the acquired data necessitates a considerable investment of time and laborious manual work. Therefore, [87] presents a new approach for automatically identifying and tracking individual yeast cells in time-lapse microscopy. The authors developed a software tool that generates synthetic images of budding yeast cells, which can be employed to train a convolutional neural network (Mask R-CNN) for instance segmentation. The synthetic images are designed to mimic brightfield images of yeast cells and are used to bypass the laborious process of manually annotating large datasets. The authors additionally employed a DBSCAN algorithm to monitor the segmented cells throughout the various frames of the microscopy movie. The combination of Mask R-CNN with DBSCAN yielded outcomes equivalent to the current state-of-the-art instrument in the domain, YeaZ. The utilization of artificial data in the advancement of CNN-based instruments for the observation of budding yeast can result in the generation of more potent, broadly applicable, and user-friendly image-processing instruments for this particular microorganism.

Having discussed the use of real and synthetic cell data in different machine learning methods, it is important to further explore the issues related to cell movement, division, aging, and migration, among others. The authors of [88] present a method that combines DRL with an agent-based modeling (ABM) framework to model cell movement in the early stage of C. elegans embryogenesis. The ABM framework is used to depict basic cell behaviors, including cell fate, division, and migration in wild-type C. elegans. The study focuses on modeling single cell movement, and the authors employ the phrases “migration cell” and “environment cell” to differentiate the cell that acquires knowledge of its migratory route from those cells that rely on the dataset of observations to navigate. The authors also use a DQN algorithm to acquire knowledge of the best path for cell migration under certain regulatory processes. The objective of the investigation is to furnish a novel instrument for investigating extensive datasets produced by real-time imaging and to acquire a more comprehensive comprehension of cellular processes and behavior.

To continue discussing the cell movement problems, the article in [89] presents a new method for understanding cell–cell interactions and collective cell behaviors in tissue development using 3D time-lapse images. This method utilizes HDRL, a technique known for its ability to learn at multiple scales and handle large amounts of data, to analyze cell movements and infer underlying biological mechanisms. The HDRL is divided into two levels, a lower level where a CNN extracts features from the environment of the migrating cell to examine the images, and a higher level where the extracted features are used to form a policy network that guides the migration cell. This method is implemented in the study of C. elegans embryogenesis, where it elucidates a multiphase and modular structure of cellular locomotion, which is confirmed by additional cellular markers. The approach generates a transferable framework that effectively distinguishes sequential migration based on rosettes from alternative methods. HDRL is verified to be an effective tool for creating models of dynamic cellular activity that can be learned from minimal input data and rules. It can also be used to uncover new characteristics of cells and tissues without prior knowledge.

As seen above, cell biology, microbiology, and artificial intelligence are interdisciplinary fields that can be combined to offer valuable assistance in experiments and studies involving cell cultures. The objectives of this study focus on the development of a methodology for a hybrid system that can be used in a number of areas of cellular and microbiological research. Such areas could be related to crop production, food industry, pharmaceutical research or patient care. Experiments on cell or tissue cultures are frequently conducted, and they can be automated by using AI technology. An AI agent can perform interventions on the cell culture under investigation through a microscope coupled to a robotic perfusion system.

## 6. Challenges, Conclusions, and Future Directions

This paper explores the significance of reinforcement learning (RL) in the realms of robotics and healthcare, considering various criteria. The discussion commences with a fundamental RL overview, elucidating the Markov Decision Process and comprehensively covering RL aspects, distinguishing between model-based and model-free, value-based and policy-based, and on-policy and off-policy approaches. This study delves deeply into RL algorithms, presenting a comprehensive overview of dynamic programming (DP), Monte Carlo (MC), and temporal difference (TD), including its two approaches, SARSA and Q-learning. Furthermore, a thorough comparison of RL algorithms is provided, summarizing their characteristics and delineating differences based on criteria such as bias, variance, computational cost, and convergence.

This systematic review then turns to RL applications in both robotics and healthcare fields. In robotics, the focus is on object grasping and manipulation, crucial across various domains, from industrial automation to healthcare. In contrast, the healthcare sector tackles cell growth and culture issues, which have garnered increasing attention in recent years, significantly contributing to modern life science research. These applications are indispensable for investigating new drug candidates, toxicological characterization of compounds, and studying a broad spectrum of biological interactions through laboratory-cultured cells. For both applications, this review analyzes the most recent influential papers, assessing their methods and results, and discussing the challenges and limitations encountered. This comprehensive and systematic review of reinforcement learning in the fields of robotics and healthcare serves as a valuable resource for researchers and practitioners, expediting the formulation of essential guidelines.

Besides what has been mentioned above about RL and its algorithms and applications, RL still faces several technical challenges in both applications discussed in Section 4. These challenges hinder the development of algorithms that could properly target the actual goal. Therefore, the challenges are divided into two parts based on each application. Robotic grasping and manipulation have many key challenges, including dexterity and control, sample efficiency, sparse rewards, and sim-to-real transfer policies [12,13,90,91,92].

The dexterity and control challenge in RL grasping tasks consists of how to address the complexity of enabling a robotic system to manipulate with finesse, precision, and adaptability [93,94,95]. The ability to alter the placement and alignment of an item, moving it from its original location to a different one, can be described as dexterity manipulation [93]. Therefore, this challenge includes several components, such as fine motor skills that enable the control of the robotic fingers or gripper with high precision. This capability is a real challenge for performing delicate movements to grasp objects of varying shapes and sizes [96]. This leads to adaptability to the variations in an object’s shape, size, weight, and material properties; therefore, the robot needs to adapt its grasping strategy to handle this diversity [68]. Moreover, the robotics system control has to balance between trajectory control and force control, where each type has its properties and goals. For more detail in this part, we refer to [93].

Another challenge is sample efficiency, considered a critical step toward learning effective grasping strategies. In other words, sample efficiency represents the ability of RL algorithms to acquire a good policy with as few samples as possible [90]. However, collecting these samples can be resource-intensive and time-consuming, even though it improves the success rate [12]. Sample efficiency encompasses several factors in achieving it in grasping tasks, such as high-dimensional state and action spaces [97], safety concerns [90], cost of exploration [98], and the simulation and real-world environment gap [99]. High-dimensional state and action spaces refer to the state spaces in the robot’s joint angles, object positions, and other environmental variables. Simultaneously, the action space refers to the actions that the robot takes and the exploration step that could lead to inefficiency in sample usage, which is itself considered a complex task. For safety concerns, which could involve objects or the robot itself, avoiding damages is crucial, limiting the number of samples that can be collected. Moreover, the disparity between the simulation and the real-world environment remains a challenge, and most recent studies have faced this issue [75,76,77]. The samples used in the simulation environment could lead to a good policy that may not transfer well to the real world due to variations in the samples, necessitating the collection of more samples for fine-tuning.

As mentioned in Section 4.2, the reward function constitutes a fundamental component of the reinforcement learning formulation, which evaluates the agent’s actions and can provide positive or negative rewards. Therefore, reward design is a crucial challenge for robotic grasping tasks, guiding the learning agent in acquiring effective grasping policies [12,90,97]. Rewards can be issued at the end of each time step (called a dense reward) [100,101], or at the end of each episode (called a sparse reward) [102,103]. Usually, grasping tasks involve sparse rewards, posing a challenge in determining which actions contribute to successful grasps. Simultaneously, the reward function must balance between exploration and exploitation, as the agent needs to explore novel actions while considering actions proven to be effective. Moreover, the reward function must consider safety issues by avoiding actions that may lead to collisions and discouraging actions that could damage the robot or objects. Therefore, all these reasons may lead to slow learning and facing many difficulties in generalizing grasping strategies among different objects. For more information, please refer to [90,97].

Last but not least, the sim-to-real transfer challenge in RL for robotics grasping tasks refers to the complexity of effectively applying policies learned in simulation environments to real-world environments [12,99,104]. Even though the simulation environment facilitates the acceleration of the training process, the real challenge is ensuring that the policies learned can generalize and perform well when deployed on real robotic systems [105]. Several key challenges are associated with sim-to-real transfer policies in RL with robotics grasping, including the reality gap, sample efficiency, and sensor mismatch [99]. Concerning the reality gap, differences between the simulation and real-world environments, such as variations in object shapes, sizes, and textures, may lead to a reality gap between the simulation and the real-world environment. Sample efficiency has been discussed above. Furthermore, sensor mismatch refers to the ability of simulated sensors to imitate the noise and characteristics of real-world sensors, which may lead to difficulties in adapting the policy obtained in a simulation environment to be transferred into a real-world environment. For more information, we refer to [99].

On the other hand, cell growth and culture issues face similar challenges as discussed above in terms of dexterity and control, sample efficiency, sparse rewards, and sim-to-real transfer policies. Regarding the limitations in the recent studies that focused on this topic, as we mentioned in Section 5.2, these challenges remain unsolved and need further investigations, particularly in collecting the data and sim-to-real transfer.

Finally, most crucially, and based on the findings of this study, there are various future research recommendations for both applications. First, the enhancement of sample efficiency is paramount due to the fact that most of the reinforcement learning algorithms necessitate more samples to acquire a specific task. Therefore, one of the main future directions is to develop algorithms that work with fewer samples. Second, the issue of real-time control arises as a major concern since most reinforcement learning algorithms reveal a noticeable lag for real-time controls [13]. Therefore, working towards enhancing the acceleration of these algorithms will enable their seamless utilization in both applications easily. Third, more than one algorithm or strategies of reinforcement learning need to be integrated to handle varying levels of uncertainty and noise in sensory data. This may lead to a robust algorithm that could overcome these problems by using hierarchical reward shaping, adaptive learning, and transfer learning problems.

## Figures and Tables

**Table 1:** Comparisons with existing reviews.

Ref.	Type of Paper	Year Coverage	Databases Used	Taxonomy-Based	Integrated Using RL Algorithms and Applications (Robotics and Healthcare)
[12]	Review paper	2016 to 2020	WoS, SD, IEEEXplore, and arXiv	✓	×
[13]	Survey	2015 to 2022	Google Scholar, IEEE Xplore, or arXiv	✓	×
[14]	Survey	1970 to 2020	N/A	×	×
[15]	Survey	1957 to 2019	N/A	✓	×
Our review paper	A comprehensive and systematic review	2021 to 2023	(SD), (IEEE), (WoS), Scopus, and others	✓	✓

**Table 2:** Statistical representation of edge weights.

Node1	Node2	Edge Weight
reinforcement learning	learning	High
learning	algorithms	High
learning	methods	High
algorithms	methods	High
grasping	manipulation	High
manipulation	robotic	High
deep learning	policy	High
policy	reward	High
reinforcement learning	algorithms	Moderate
reinforcement learning	methods	Moderate
reinforcement learning	control	Moderate
learning	control	Moderate
algorithms	control	Moderate
methods	control	Moderate
grasping	robotic	Moderate
deep learning	reward	Moderate

**Table 3:** Comparison of DP, MC, and TD algorithms.

Algorithm	Model-Based/Model-Free	Requires Model	Bias	Variance	On/Off-Policy	Computational Cost	Convergence
DP	Model-Based	Yes	High	Low	On-Policy	High	Faster than MC and TD
MC	Model-Free	No	Low	High	On-Policy	Medium	Slower than DP and TD
TD	Model-Free	No	High	Low	On/Off-Policy	Low	Faster then MC

**Table 4:** Emergence of deep reinforcement learning algorithms.

Year	Algorithm’s Title	Description	Ref.
2013	Deep Q Learning Network (DQN)	DQN is one of the pioneering deep reinforcement learning (DRL) algorithms that utilizes Q-learning and convolutional neural networks (CNNs) to learn control policies by processing vast amounts of high-dimensional data. This algorithm was implemented in the context of seven Atari 2600 games, demonstrating superior performance compared to the existing methods and even surpassing human proficiency in half of the games. Additionally, this algorithm can handle continuous states and discrete actions.	For more detailed information, we refer to [60].
2014	Deterministic Policy Gradient (DPG)	DPG is considered the first algorithm in RL designed to handle continuous action spaces. The estimation of the gradient of the action-value function is conducted deterministically, leading to enhanced computational efficiency compared to stochastic policy gradients.	For more detailed information, we refer to [61].
2015	Deep Deterministic Policy Gradient (DDPG)	DDPG is an actor-critic algorithm designed to tackle challenges pertaining to continuous control problems in RL. This algorithm was based on DPG and has successfully addressed various simulated physics tasks. Both DQN and DPG have suffered from high bias and high variance, but this technique has combined previous techniques, leading to reduced bias and variance.	For more detailed information, we refer to [62].
2015	Trust Region Policy Optimization (TRPO)	TRPO stands as an additional deep reinforcement learning (DRL) algorithm designed to optimize policies by ensuring a monotonic enhancement and showcasing sturdy performance across a range of tasks. This algorithm proves to be a potent technique in addressing high-dimensional continuous control predicaments.	For more detailed information, we refer to [63].
2017	Proximal Policy Optimization (PPO)	PPO is a DRL algorithm that is categorized within the realm of policy gradient techniques. In contrast to TRPO, PPO is noted for its ease of implementation, broader applicability, and superior performance in terms of sample efficiency.	For more detailed information, we refer to [64].

**Table 5:** RL methods with their actions, framework, and codes.

Ref.	Year	RL-Method	Action	Framework	Code
[68]	2022	Graph-based Q-learning model (DQN and Q-Net)	Grasping Pushing	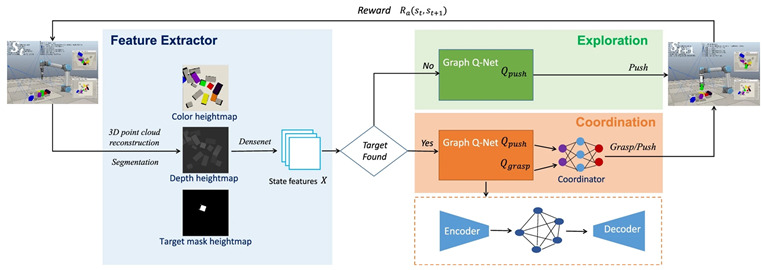	https://github.com/ttongjiayuan/the-dataset-of-grasping-occluded-objects (accessed on 5 July 2022)
[69]	2022	Viewpoint Adjusting and Grasping Synergy (VAGS) strategy based on deep reinforcement learning (DRL)	Viewpoint Adjusting Grasping	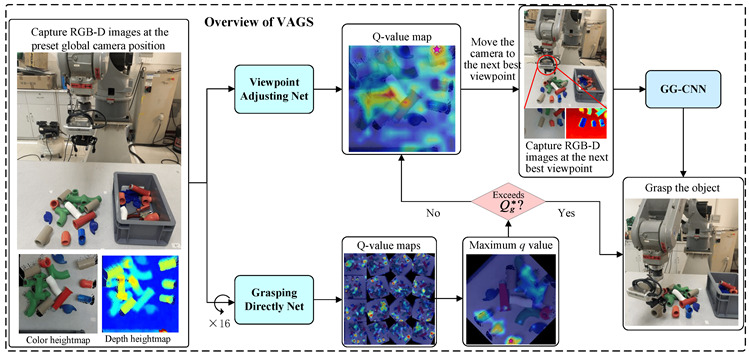	N/A
[70]	2022	Proximal Policy Optimization (PPO) and Soft Actor-Critic (SAC)	Grasping Lifting	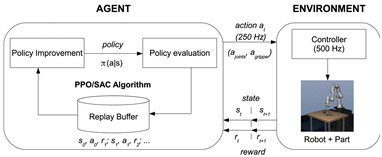	https://github.com/Asad-Shahid/Intelligent-Task-Learning (accessed on 13 April 2022)
[71]	2022	DDPG-Sparse + SLDR	Pick and Place	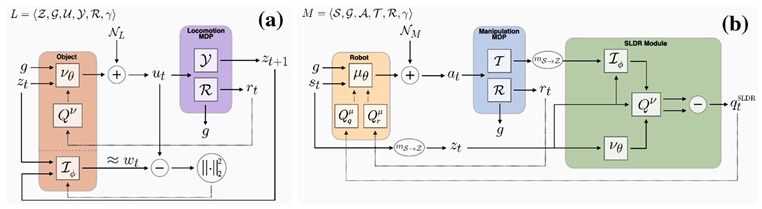	1- Source code (https://github.com/WMGDataScience/sldr) (accessedon 30 October 2019) 2- Environments Code (https://github.com/WMGDataScience/gym_wmgds) (accessed on 30 October 2019)
[72]	2022	Dueling Deep Q-learning Network (DDQN)	Grasping	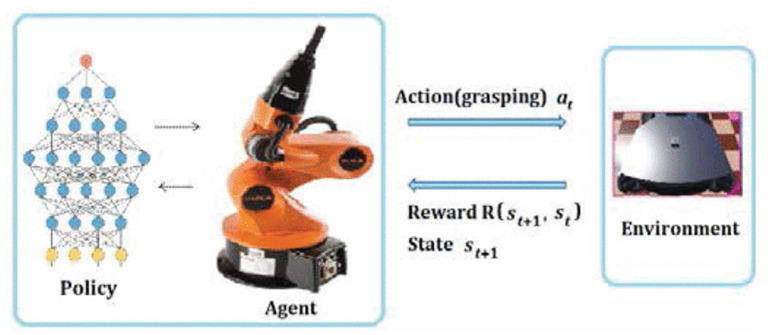 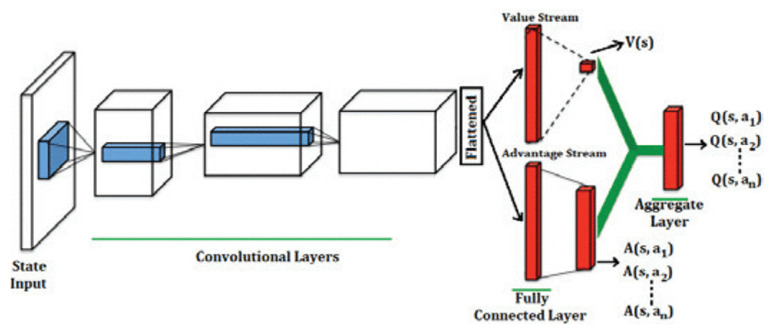	N/A
[73]	2023	You Only Look Once (YOLO) algorithm and Soft Actor-Critic (SAC) algorithm	Grasping/Pick and Place	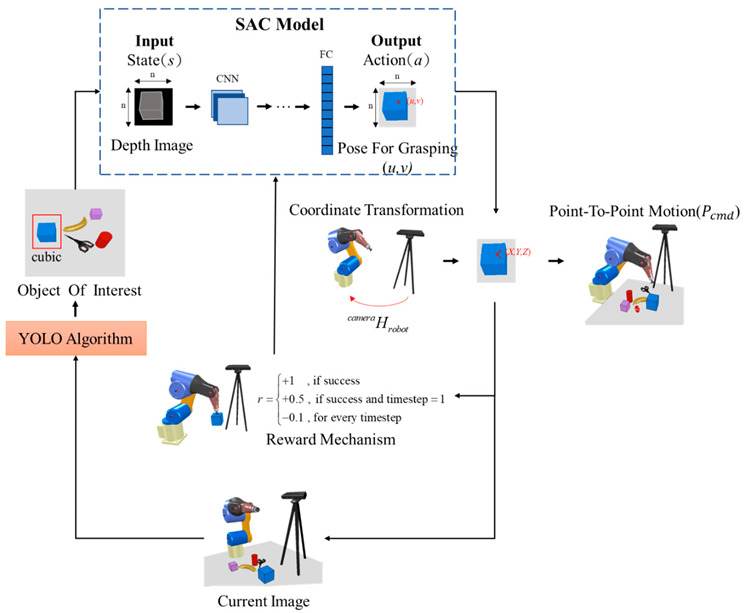	N/A
[74]	2023	Q Mixing Network with Planar and Spherical Affordances (QMIX-PSA)	Grasping	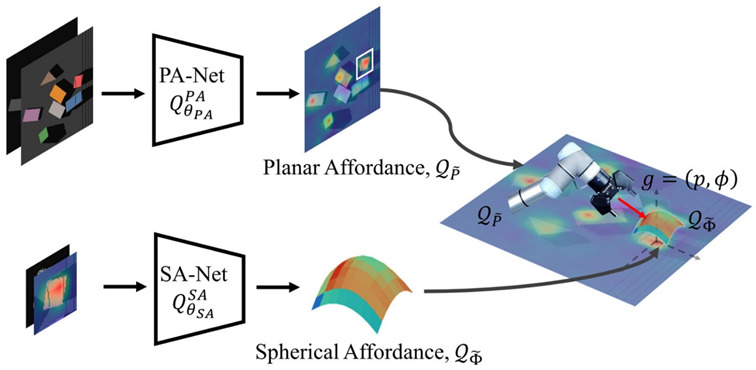	N/A
[75]	2023	Grasp Pose is All You Need (G-PAYN) with the Soft Actor-Critic (SAC) algorithm	Grasping	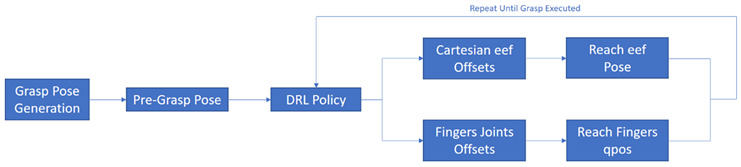	https://github.com/hsp-iit/rl-icub-dexterous-manipulation (accessed on 25 January 2022)
[76]	2023	deep Q-network (DQN)	Pick-and-Place Prehensile (grasping) and non-prehensile (left-slide and right-slide)	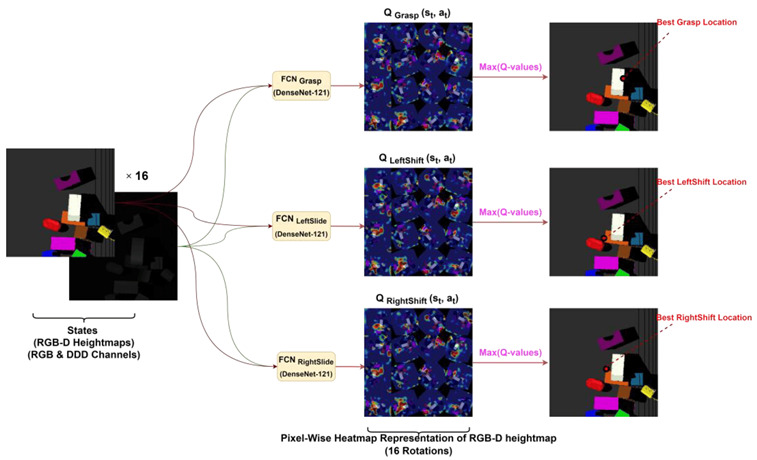	N/A
[77]	2023	Soft Actor-Critic (SAC) and Proximal Policy Optimization (PPO)	Reach, Grasp, and Pick-and-Place	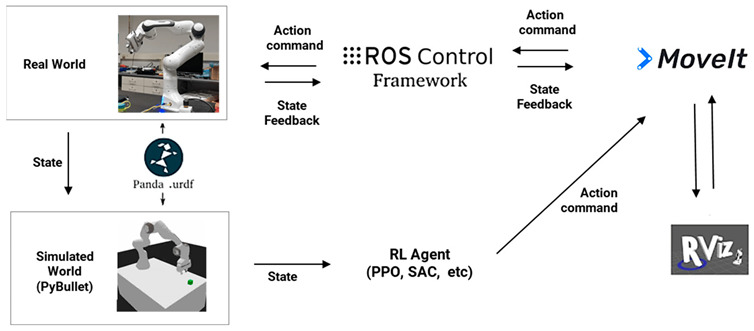	N/A

**Table 6:** RL methods with their results and limitations.

Ref.	Year	RL-Method	Metrics	Results	Limitations
[68]	2022	Graph-based Q-learning model (DQN and Q-Net)	1- SR (%) Success Rate 2- MN (Motion Number)	1- In the simulation experiment: exploration task using a dataset of 10 different scenes, SR = 100% with an average of MN of less than 2); coordination task, SR = 91% with an average of MN = 3.2. 2- In the simulation experiment, coordination task with dataset extended to 20 different coordination scenes, SR = 95% with an average of MN = 2. 3- Real robot experiment: SR = 91% with an average of MN = 7.3.	1- The model works on block shape objects only and has not generalized to all target objects.
[69]	2022	VAGS strategy based on DRL	1- Grasp Success Rate (GSR) 2- Scene Clearing Rate (SCR) 3- Motion Number 4- Mean Picks Per Hour	1- In the simulation experiment of 1 to 10 objects, the GSR = 83.50%, and the SCR = 95%. 2- In the simulation experiment with application of DAES, the results, with and without VANet, showed an average grasp success rate improvement (w/o VANet = 76.38%, w/VANet = 86.87%) and SCR improvement (w/o VANet = 84%, w/VANet = 95%). 3- In real-world experiments, the results showed a (GSR of 83.05%, an average grasping time of 8.5 s, a mean picks per hour of 348, and a motion number of 1.38).	The use of high-degree-of-freedom (DOF) grasping scenarios is suggested as a future avenue for enhancing grasping stability by achieving six-DOF grasping.
[70]	2022	PPO and Soft SAC	1- Success Rate	1- In the simulation experiment, the success rate was 100%. 2- In the real-world experiment using the Franka Emika Panda robot, the success rate was 100%.	1- It is important to highlight that the methodology is assessed on a grasping assignment that encompasses a Franka Emika Panda manipulator. Although the authors propose that the acquisition can be generalized to diverse geometric forms and dimensions, slight alterations in the object’s position, as well as different initial configurations of the robot, may impact the results. Therefore, additional investigation and experimentation are required to validate this assertion. 2- Moreover, the suggested technique is assessed primarily by considering the success rate of grasping tasks, and its performance in other manipulation tasks remains unclear. 3- Furthermore, the article lacks a comparison of the suggested strategy with other state-of-the-art methods for robotic grasping. Therefore, this could provide further information about its strengths and weaknesses.
[71]	2022	DDPG-Sparse+SLDR	1- Success Rate (SR)	The proposed approach has achieved a SR of 100% over 13 tasks of increasing complexity.	1- The first limitation arises when the simulated locomotion demonstrations (SLDs) are less effective when the ideal object locomotion becomes increasingly dependent on the actions performed by the robot. 2- The second limitation is the prerequisite for a simulated environment to apply the proposed methodology effectively. 3- The third potential limitation of using simulated locomotion as auxiliary rewards is that it may not apply to certain tasks. In tasks involving the manipulation of a pen using Shadow’s hand, a challenge arises when attempting to replicate a specific pen movement without dropping it. Despite the pen’s ability to rotate and translate itself to facilitate locomotion tasks, this predicament has the potential to undermine the effectiveness of the manipulation strategy, regardless of the acquisition of an optimal locomotion policy.
[72]	2022	Dueling Deep Q-learning Network(DDQN)	N/A	The proposed model has exhibited superior results when the batch size is set to 16, particularly in terms of immediate reward values.	There are several limitations to the proposed model: 1- The model exclusively addresses the grasping of solid and rigid objects, without considering variations in object types. 2- Throughout the evaluation process, the location and shape of the target objects remained constant, implying that the proposed model was tested under unchanging conditions. 3- The proposed model has only been employed within a simulation environment.
[73]	2023	YOLO algorithm and SAC algorithm	1- Training time 2- Number of graspingattempts 3- Rate of successful grasping	1- In a simulation environment, the suggested technique (transfer learning + YOLO + SAC) effectively reduced the training time to 6443 s, while without using the approach was 15.9 times longer. At the same time, the number of grasping was 1323 attempts using the proposed approach, while without it was 28.8 times larger. 2- In a real-world environment, the proposed approach demonstrated a successful grasping rate for various objects: 19 out of 20 for building blocks, 6 out of 10 for apples, 6 out of 10 for bananas, 8 out of 10 for oranges, and 9 out of 10 for cups. It is worth noting that apples and oranges were not part of the training set.	One limitation of this approach is its dependence on smooth object surfaces for successful grasping using the suction nozzle. As a result, the noticeable variations between the simulated and the real-world environment experimental setup lead to a lower success rate when attempting to grasp bananas due to surface variations between the two environments.
[74]	2023	QMIX-PSA	1- The grasp success rate (GSR) 2- The average grasp quality (AGQ)	The experiment was conducted in a real-world environment using a UR3 robot. The study employed twenty metal workpieces and sixteen daily items with various complex shapes were used in the study. The proposed method was tested in three distinct scenarios: single objects, scattered objects, and cluttered objects. The results are as follows: 1- When comparing 6DGL with its six peers across these three scenarios, the results show that 6DGL outperforms most of its counterparts. Notably, in the cluttered scenario, 6DGL achieved a 0.82 and 0.83 Grasping Success Rate (GSR) when grasping metal workpieces and daily items, respectively. 2- The results for 6DGL show an Average Grasp Quality (AGQ) of 0.67 for metal workpieces and 0.75 for daily items, indicating a certain level of robustness. 3- When presented with 16 unseen objects in a cluttered environment, 6DGL achieved a GSR of 0.77 and an AGQ of 0.68, outperforming most competitors.	There are a few limitations to this study, including: 1- It is important to note that the experiments were conducted in a controlled environment, and it remains uncertain how well the approach would perform in more complex and dynamic real-world scenarios. 2- Furthermore, the proposed approach relies on high-dimensional RGB-D data as input, which may not be available or feasible in all robotic grasping applications.
[75]	2023	G-PAYN with SAC algorithm	1- Success Rate (SR) 2- Execution Time (ET)	In a simulation environment, the proposed method G-PAYN has outperformed more than half of the experiments in terms of success rate by a significant margin (0.3 and 0.15 gap) and achieved equivalent results in virtually all of the remaining cases. Additionally, G-PAYN has a faster execution time than the other DRL baselines	1- The presented method relies on automatically gathered demos and an initial grasp stance created by an external algorithm, which may not be the suitable and most effective way to learn the task. 2- The training was conducted solely in a simulation environment, and it may not perform as effectively in a real-world scenario, despite the assertion that their policies can be implemented on an actual robot without necessitating any modifications to the action and state spaces.
[76]	2023	DQN	1- Success Rate (SR) 2- Grasping Success (GS)	In a simulation environment using a V-REP simulator, the proposed approach, known as C&S (Grasping and Sliding), has been evaluated for its performance by comparing it with a deep learning baseline framework as follows: 1- Comparing the C&S approach with a deep-learning-only-based supervised binary classification approach. The C&S achieved a success rate of approximately 84%, while the binary classification approach attained a success rate of around 57%. 2- Comparing the C&S approach with another C&S approach that utilized the ResNet-101 architecture pre-trained on ImageNet. The original C&S approach outperformed the ResNet-based approach by a margin of approximately 13%. 3- Comparing the C&S approach with another C&S approach in which the reward allocation for non-prehensile manipulations was discontinued. Removing this component led to a performance degradation of around 22% in terms of SR. 4- Comparing the C&S approach with another C&S approach that omitted the use of depth channels. The results indicated that the SR dropped by approximately 51% over a period of 3000 episodes. 5- Finally, evaluating the performance of the original C&S approach under unseen circumstances, categorized into four scenarios: In minimum clutter, C&S achieved a success rate of 84% and a grasping success of 96%. In medium clutter, the success rate was 82%, with a grasping success of 95%. In maximum clutter, C&S achieved a success rate of 74% and a grasping success of 82%. In complicated scenarios, the success rate reached 65%, with a grasping success of 73%.	The article acknowledges several limitations of the proposed approach which are: 1- The agent underwent training using a restricted set of 3D block shapes, but its potential could be augmented and expanded to encompass items frequently encountered in daily life, such as bottles, cups, and balls. 2- The concatenation factor of the feature map in DenseNet-121 could potentially be considered a limitation. 3- The approach involves only a sequential combination of robotic manipulations, and it could benefit from an increased number of actions and the introduction of parallel robotic manipulation combinations, including novel techniques like stacking, rolling, and rotating. 4- As the system scales, issues related to the overestimation of future rewards could potentially arise.
[77]	2023	SAC and PPO	Success Rate (SR)	The simulation environment has been divided into three parts: “Panda reach and Panda grasp with dense rewards”: Both PPO and SAC achieved a 100% SR on the reach task. For the grasp task, PPO achieved an 89% success rate, while SAC achieved a 92% success rate. “Panda reach and Panda grasp with sparse rewards”: Both PPO and SAC achieved a 100% SR on the reach task. For the grasp task, PPO achieved a 90% success rate, while SAC achieved a 95% success rate. “Panda pick-and-place with dense rewards”: PPO achieved an 85% success rate, while SAC achieved a 71% success rate. In the real-world environment, which has been divided into two parts: “Panda reach and Panda grasp with dense rewards”: Both PPO and SAC achieved a 90% SR on the reach task.For the grasp task, PPO achieved a 70% success rate, while SAC achieved an 80% success rate. “Panda pick-and-place with dense rewards”: PPO achieved a 70% success rate, while SAC achieved a 60% success rate.	There are several limitations to the proposed model: 1- Differences in geometry between the simulation and real-world environments could necessitate modifications to the target objects to achieve a better match. 2- Not implementing a more effective sim-to-real transfer method resulted in high computational costs and reduced environmental accuracy. 3- The absence of positional sensing in real-world target blocks to ensure alignment with their simulated counterparts during training tasks. 4- The authors did not compare the use of these algorithms to other RL algorithms or traditional control strategies. 5- The approach employed a single robot arm for reach, grasp, and pick-and-place tasks. While the results are promising, it remains unclear how well the approach would generalize to other types of robots or tasks.

## Data Availability

Not applicable.

## Supplementary Materials

The following supporting information can be downloaded at: https://www.mdpi.com/article/10.3390/s24082461/s1.
