# Supplementary material for: Reinforcement Learning Algorithms and Applications in Healthcare and Robotics: A Comprehensive and Systematic Review
Source: Sensors (Basel). 2024 Apr 11;24(8):2461. doi: 10.3390/s24082461 (PMC11053800; doi:10.3390/s24082461)
Supplement: Supplementary file 1 [file sensors-24-02461-s001.zip › sensors-2920523-supplementary file.pdf]

# **Supplementary material** for Reinforcement Learning Algorithms and Applications in Healthcare and Robotics: A Comprehensive and Systematic Review

**Mokhaled N. A. Al-Hamadani [1,2,3,*], Mohammed A. Fadhel [4], Laith Alzubaidi [4,5,6] and Harangi Balazs [1]**

[1] Department of Data Science and Visualization, Faculty of Informatics, University of Debrecen, H-4032 Debrecen, Hungary; harangi.balazs@inf.unideb.hu

[2] Doctoral School of Informatics, University of Debrecen, H-4032 Debrecen, Hungary

[3] Department of Electronic Techniques, Technical Institute/Alhawija, Northern Technical University, Adan, 36001 Kirkuk, Iraq

[4] Research and Development Department, Akunah Company, Brisbane, QLD 4120, Australia; mohammed.a.fadhel@uoitc.edu.iq (M.A.F.); l.alzubaidi@qut.edu.au (L.A.)

[5] School of Mechanical, Medical, and Process Engineering, Queensland University of Technology, Brisbane, QLD 4000, Australia

[6] Centre for Data Science, Queensland University of Technology, Brisbane, QLD 4000, Australia

* Correspondence: alhamadani.mokhaled@inf.unideb.hu

This document includes all the framework figures that have been used in Table 5. These figures are high-resolution and more readable now.

## **1. Supplementary figures**

The framework figure for reference [68] is shown below:

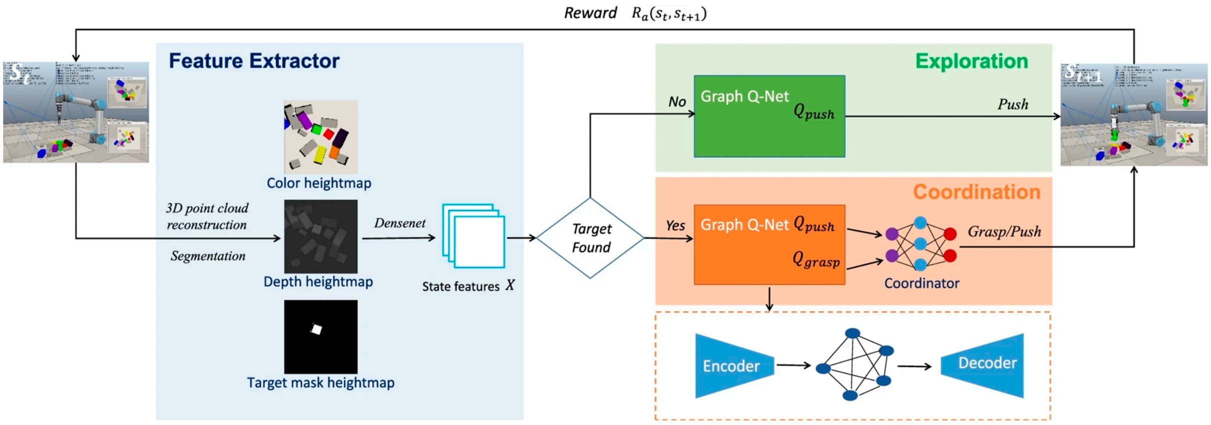


The framework figure for reference [69] is shown below:

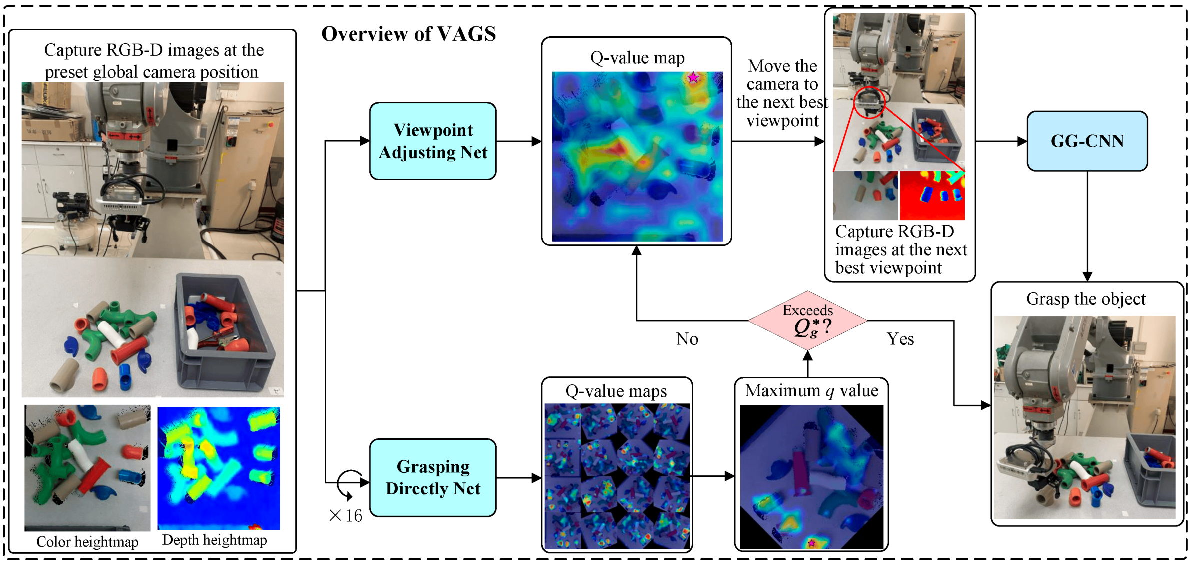


The framework figure for reference [70] is shown below:

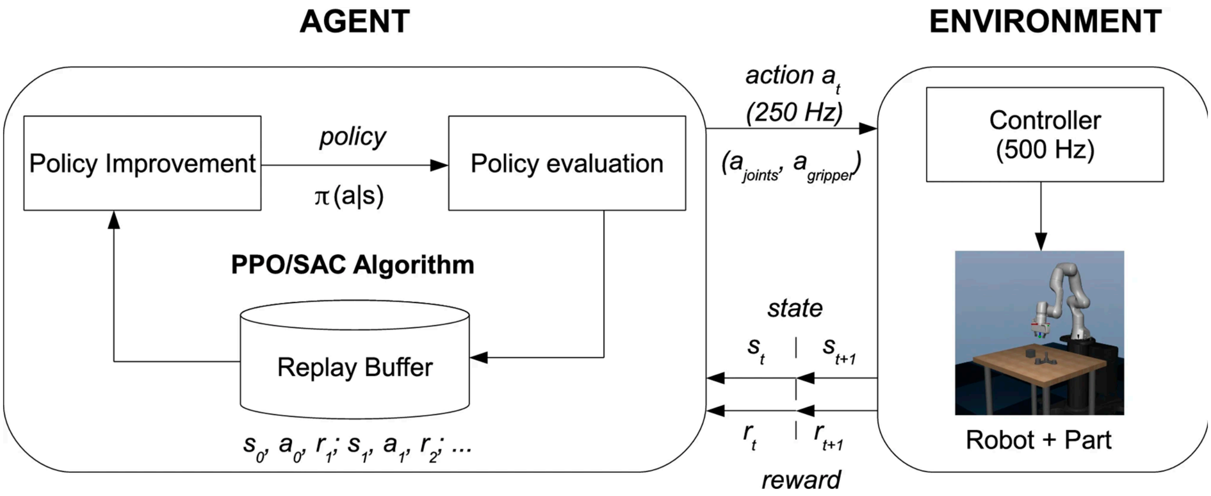


The framework figure for reference [71] is shown below:

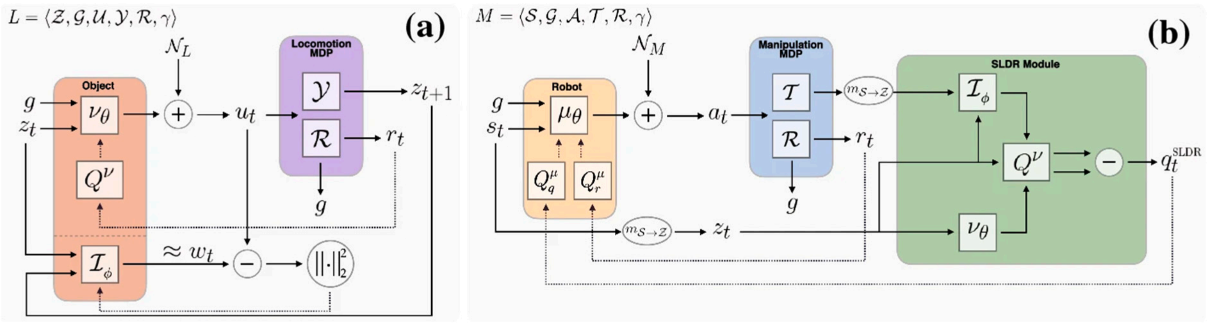


The framework figure for reference [72] is shown below:

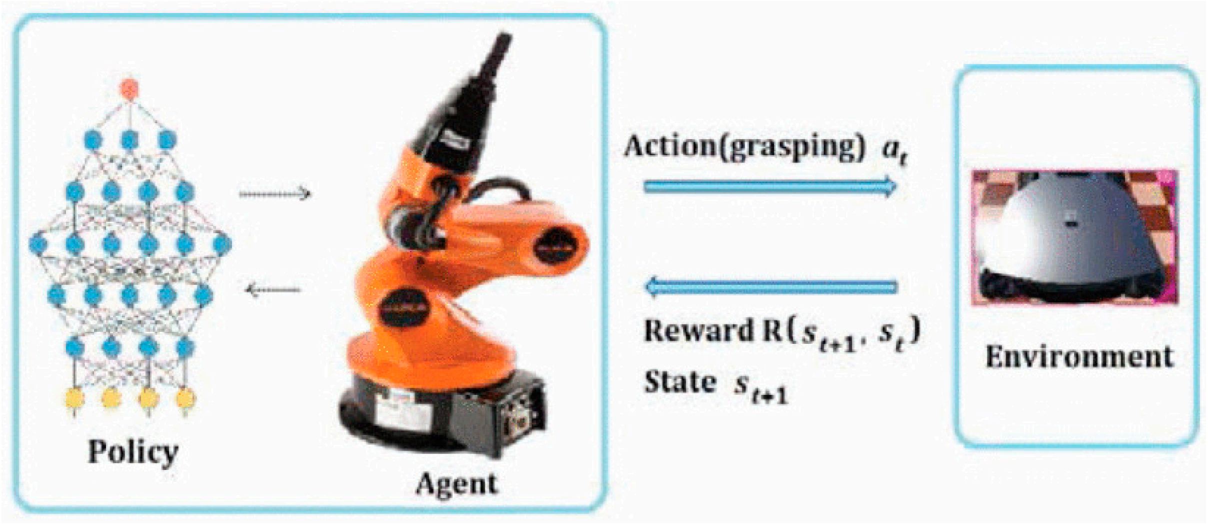


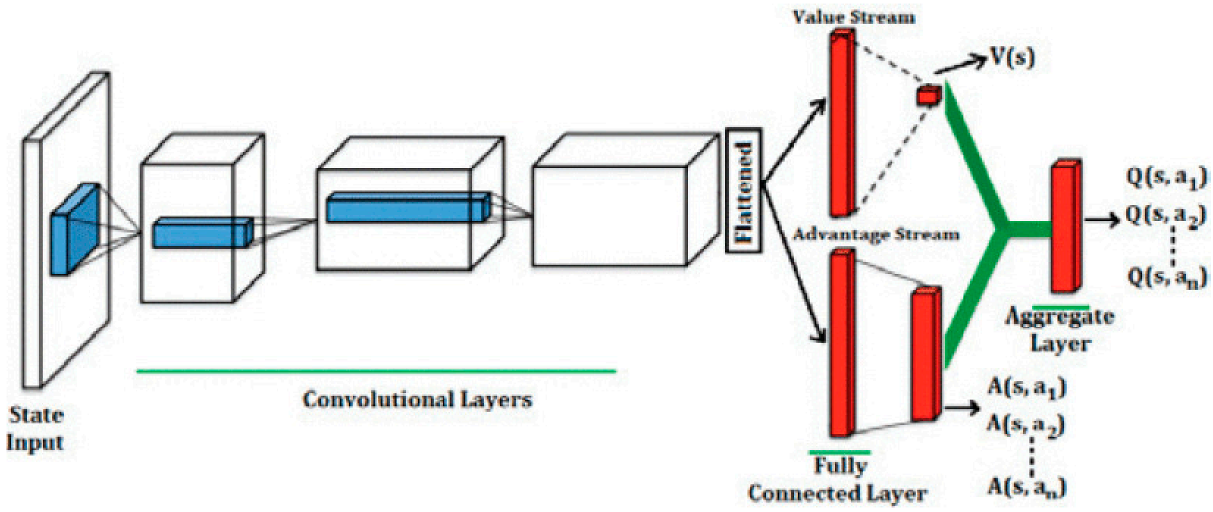


The framework figure for reference [73] is shown below:

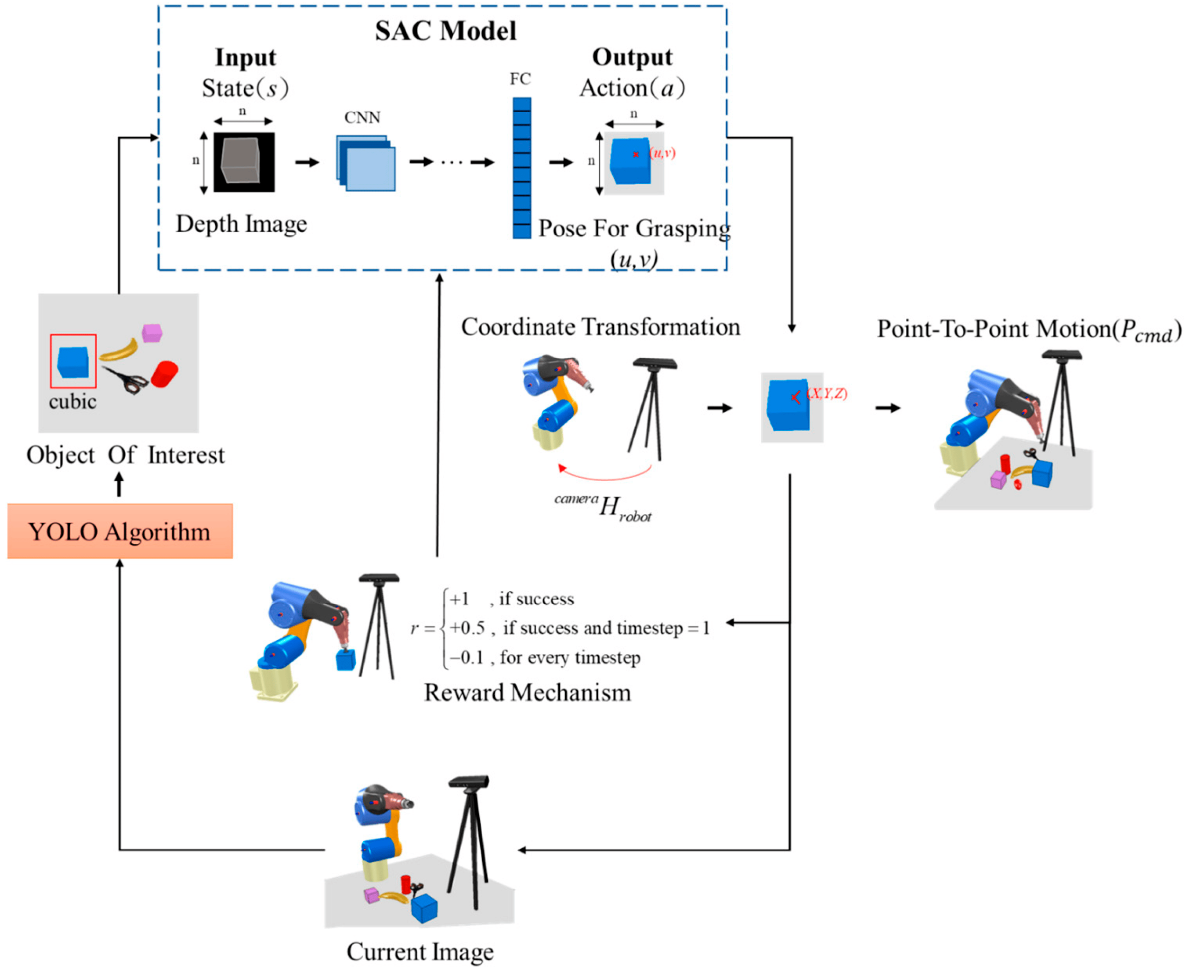


The framework figure for reference [74] is shown below:

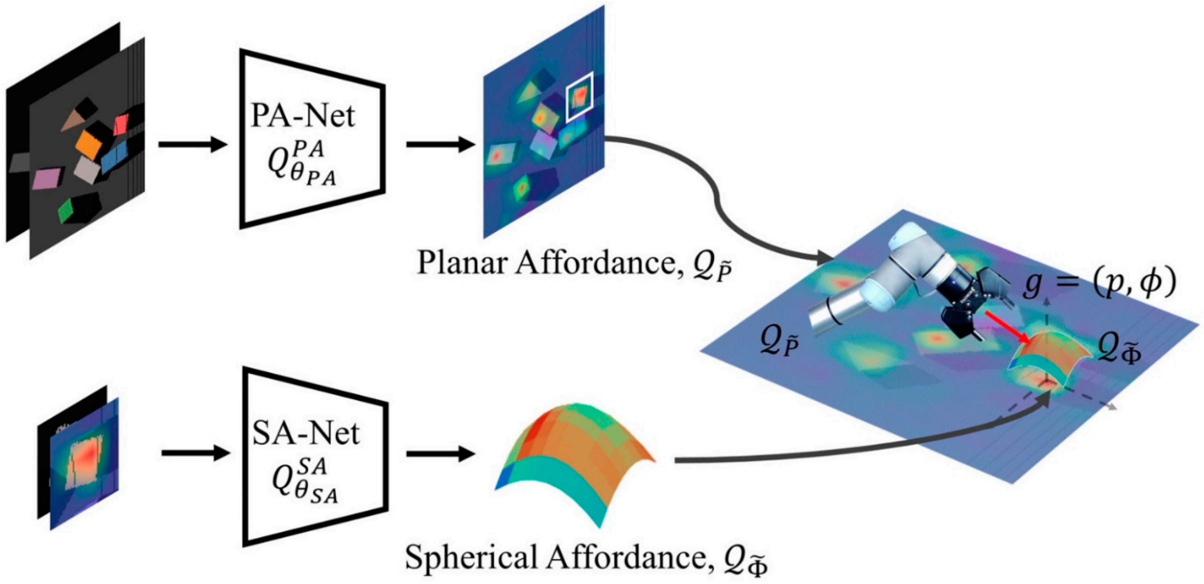

The framework figure for reference [75] is shown below:

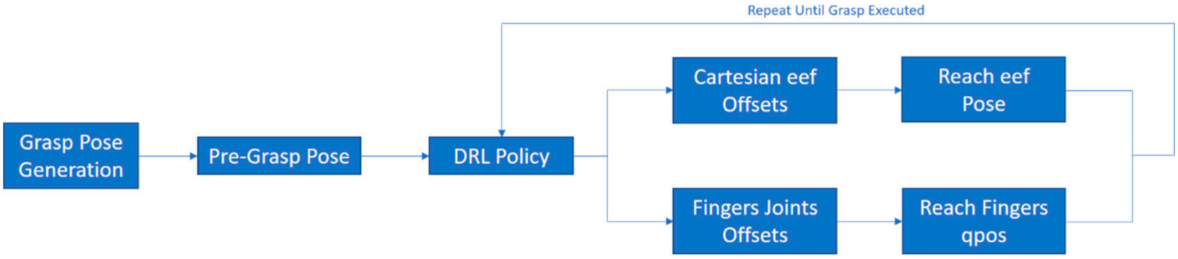


The framework figure for reference [76] is shown below:

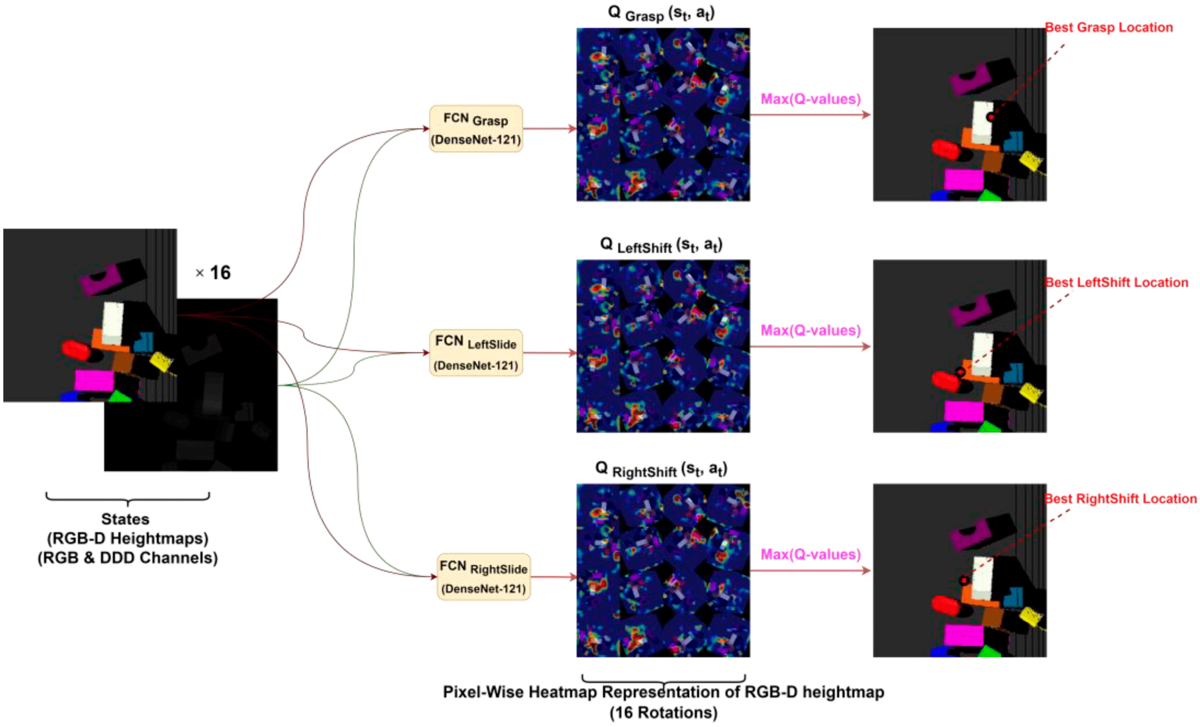

The framework figure for reference [77] is shown below:

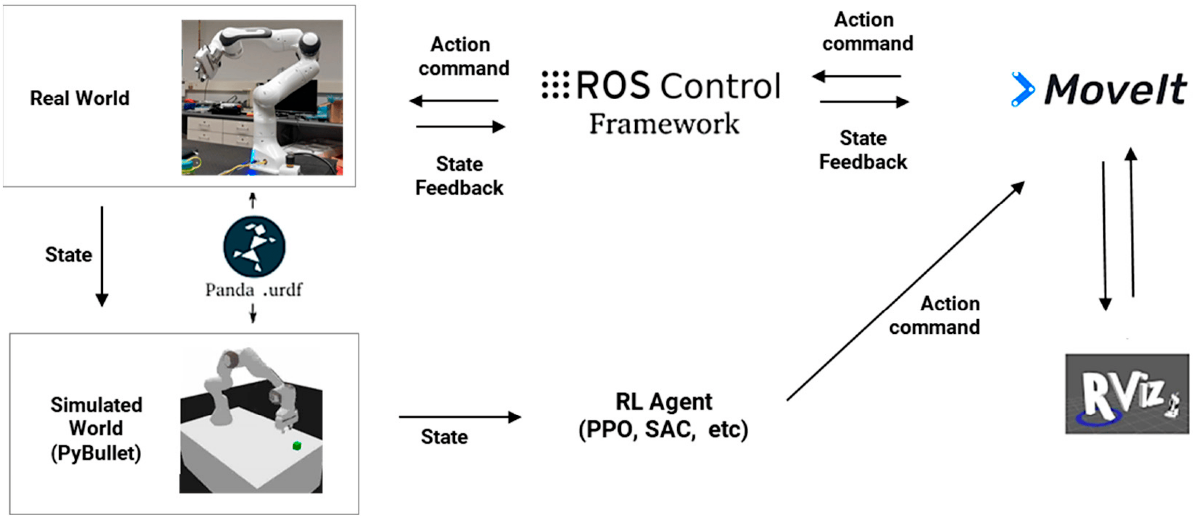


# References


68. G. Zuo, J. Tong, Z. Wang and D. Gong, "A Graph-Based Deep Reinforcement Learning Approach to Grasping Fully Occluded Objects," Cognitive Computation , vol. 15, p. pages36–49, 2022.
69. N. Liu, C. Guo, R. Liang and D. Li, "Collaborative Viewpoint Adjusting and Grasping via Deep Reinforcement Learning in Clutter Scenes," Machines, vol. 10, no. 12, 2022.
70. A. A. Shahid, D. Piga, F. Braghin and L. Roveda, "Continuous control actions learning and adaptation for robotic manipulation through reinforcement learning," Autonomous Robots, vol. 46, no. 3, p. 483–498, 2022.
71. O. Kilinc and G. Montana, "Reinforcement learning for robotic manipulation using simulated locomotion demonstrations," Machine Learning, vol. 111, p. 465–486, 2022.
72. M. Coskun, O. Yildirim and Y. Demir, "Robotic Grasping in Simulation Using Deep Reinforcement Learning," in 7th International Conference on Computer Science and Engineering (UBMK), Diyarbakir, Turkey, 2022.
73. Y.-L. Chen, Y.-R. Cai and M.-Y. Cheng, "Vision-Based Robotic Object Grasping—A Deep Reinforcement Learning Approach," Machines, vol. 11, no. 2, 2023.
74. Y. Hou and J. Li, "Learning 6-DoF grasping with dual-agent deep reinforcement learning," Robotics and Autonomous Systems, vol. 166, 2023.
75. F. Ceola, E. Maiettini, L. Rosasco and L. Natale, "A Grasp Pose is All You Need: Learning Multi-fingered Grasping with Deep Reinforcement Learning from Vision and Touch," arXiv, 2023.
76. M. B. Imtiaz, Y. Qiao and B. Lee, "Prehensile and Non-Prehensile Robotic Pick-and-Place of Objects in Clutter Using Deep Reinforcement Learning," Sensors, vol. 23, no. 3, 2023.
77. A. Lobbezoo and H.-J. Kwon, "Simulated and Real Robotic Reach, Grasp, and Pick-and-Place Using Combined Reinforcement Learning and Traditional Controls," Robotics, vol. 12, no. 1, 2023.
